# Nahal Ein Gev II, a Late Natufian Community at the Sea of Galilee

**DOI:** 10.1371/journal.pone.0146647

**Published:** 2016-01-27

**Authors:** Leore Grosman, Natalie D. Munro, Itay Abadi, Elisabetta Boaretto, Dana Shaham, Anna Belfer-Cohen, Ofer Bar-Yosef

**Affiliations:** 1 Institute of Archaeology, Mount Scopus, The Hebrew University of Jerusalem, Jerusalem, 91905, Israel; 2 Department of Anthropology, Unit 1176, 354 Mansfield Road, University of Connecticut, Storrs, CT, 06269–1176, United States of America; 3 D-REAMS Radiocarbon Laboratory, 76100, Rehovot, Israel; 4 Department of Anthropology, Harvard University, Cambridge, MA, 02138, United States of America; ICREA at the Universitat Autònoma de Barcelona, SPAIN

## Abstract

The Natufian culture is of great importance as a starting point to investigate the dynamics of the transition to agriculture. Given its chronological position at the threshold of the Neolithic (ca. 12,000 years ago) and its geographic setting in the productive Jordan Valley, the site of Nahal Ein Gev II (NEG II) reveals aspects of the Late Natufian adaptations and its implications for the transition to agriculture. The size of the site, the thick archaeological deposits, invested architecture and multiple occupation sub-phases reveal a large, sedentary community at least on par with Early Natufian camps in the Mediterranean zone. Although the NEG II lithic tool kit completely lacks attributes typical of succeeding Pre Pottery Neolithic A (PPNA) assemblages, the artistic style is more closely related to the early PPNA world, despite clear roots in Early Natufian tradition. The site does not conform to current perceptions of the Late Natufians as a largely mobile population coping with reduced resource productivity caused by the Younger Dryas. Instead, the faunal and architectural data suggest that the sedentary populations of the Early Natufian did not revert back to a nomadic way of life in the Late Natufian in the Jordan Valley. NEG II encapsulates cultural characteristics typical of both Natufian and PPNA traditions and thus bridges the crossroads between Late Paleolithic foragers and Neolithic farmers.

## Introduction

As the last cultural entity prior to the emergence of Neolithic communities in the southern Levant, the Late Natufian phase is an important starting point for investigations of the internal dynamics of the Natufian culture and how they led to the transition to agriculture. The Natufian was first recognized and defined [1] in the Mediterranean zone of the southern Levant [2]. It’s typical characteristics emerged by 15,000 cal BP and provide an intensive archaeological signal primarily characterized by large sites, permanent architecture, established burial practices, and rich groundstone, bone tool and artistic traditions (e.g.,[3, 4]).

Ambiguities concerning Late Natufian cultural dynamics and their role in the transition to agriculture have focused on questions of site permanence, the impact of climatic change wrought by the Younger Dryas (hereafter YD) event, the pace of the transition and the role of local populations in this change. Research of these questions has focused on the Mediterranean zone, with less attention given to the Late Natufian of the Jordan Valley. Nevertheless, studies increasingly suggest that the first agricultural communities in the southern Levant emerged in the Jordan Rift Valley during the earliest Neolithic phase, the Pre-Pottery Neolithic A (hereafter PPNA; [5–9]). Understanding cultural developments in the Jordan Valley is of significance for reconstructing agricultural origins especially given that recent chronological refinements reveal a possible brief period of overlap between the Natufian and PPNA [10]; the Natufian culture is dated to 11,300 cal. BP, while the earliest PPNA sites date to ca. 11,700 cal BP. Characterizing this important period of potential overlap in the Jordan Valley is crucial for understanding the socioeconomic processes that marked the shift from Palaeolithic foragers to Neolithic agricultural communities.

**Fig 1:**
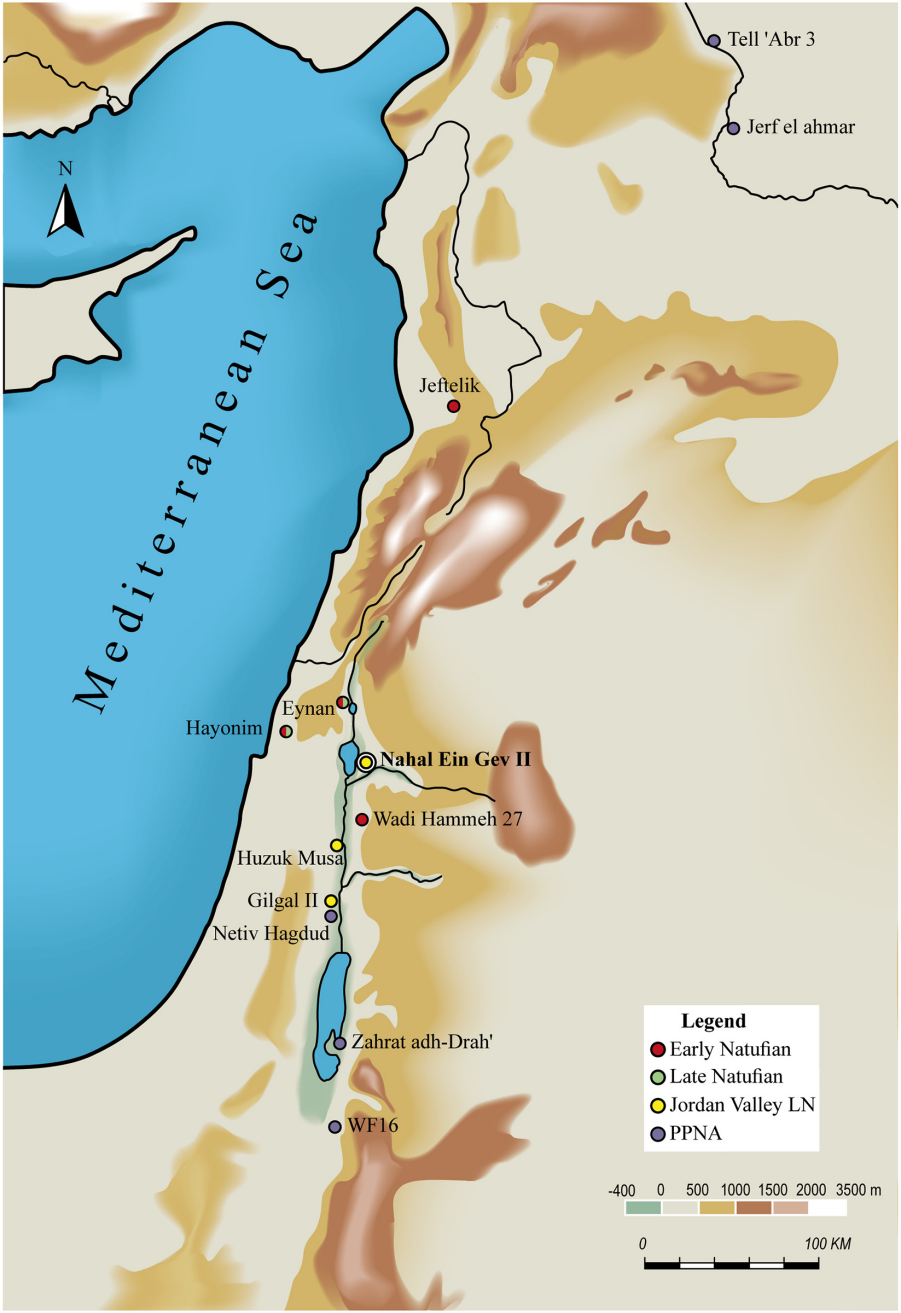
Location of NEG II in the Southern Levant and other sites mentioned in the text.

Given its chronological position and its geographic setting in the productive Jordan Valley, the Late Natufian site of Nahal Ein Gev II (hereafter NEG II) provides the opportunity to investigate several issues critical for understanding Late Natufian adaptations and their implications for the transition to agriculture. First, the late date of NEG II provides the opportunity to test whether the changes inherent in this transformation were initiated by local Natufian populations. This issue is addressed by comparing patterns in Late Natufian material culture with those of succeeding PPNA populations. Second, the location of NEG II in the Jordan Valley allows us to reconstruct local human adaptations to the YD climatic event (Fig 1). Site permanence at NEG II is investigated through architectural, lithic and faunal indicators and compared to similar indicators in the Mediterranean zone to investigate how it fits with recent interpretations of regional population mobility.

These issues are addressed by presenting preliminary findings from the first few seasons of excavation at NEG II comprising chronological, stratigraphic, architectural and human burial data as well as lithic, faunal, groundstone and art assemblages.

## Nahal Ein Gev II

NEG II is located in Nahal (wadi) Ein Gev, at the middle of a perennial stream that flows west to the Sea of Galilee (SW 261750–743280; NE 261978–743404, Fig 1). The site is situated on a flat alluvial terrace on the right bank of a prominent meander in the streambed about 2 km east of the Sea of Galilee at the Natufian times. It was briefly tested in 1973 [11]. The testing took advantage of a small modern foxhole excavated at the eastern edge of the site that exposed a *ca*. 1.2 m deep stratigraphic section.

The section of the foxhole was extended to 2.2 m below the surface during recent excavations. The stratigraphy of the pit reveals homogenous Natufian cultural remains across the full span of the section. The intensity of site occupation suggested by the thickness of these cultural deposits has no parallel in any contemporary site in the southern Levant. The stratigraphic sequence and two stone concentrations exposed in the foxhole reveal at least three phases of occupation in this part of the site, verified by the stratigraphy of other excavated areas.

**Fig 2:**
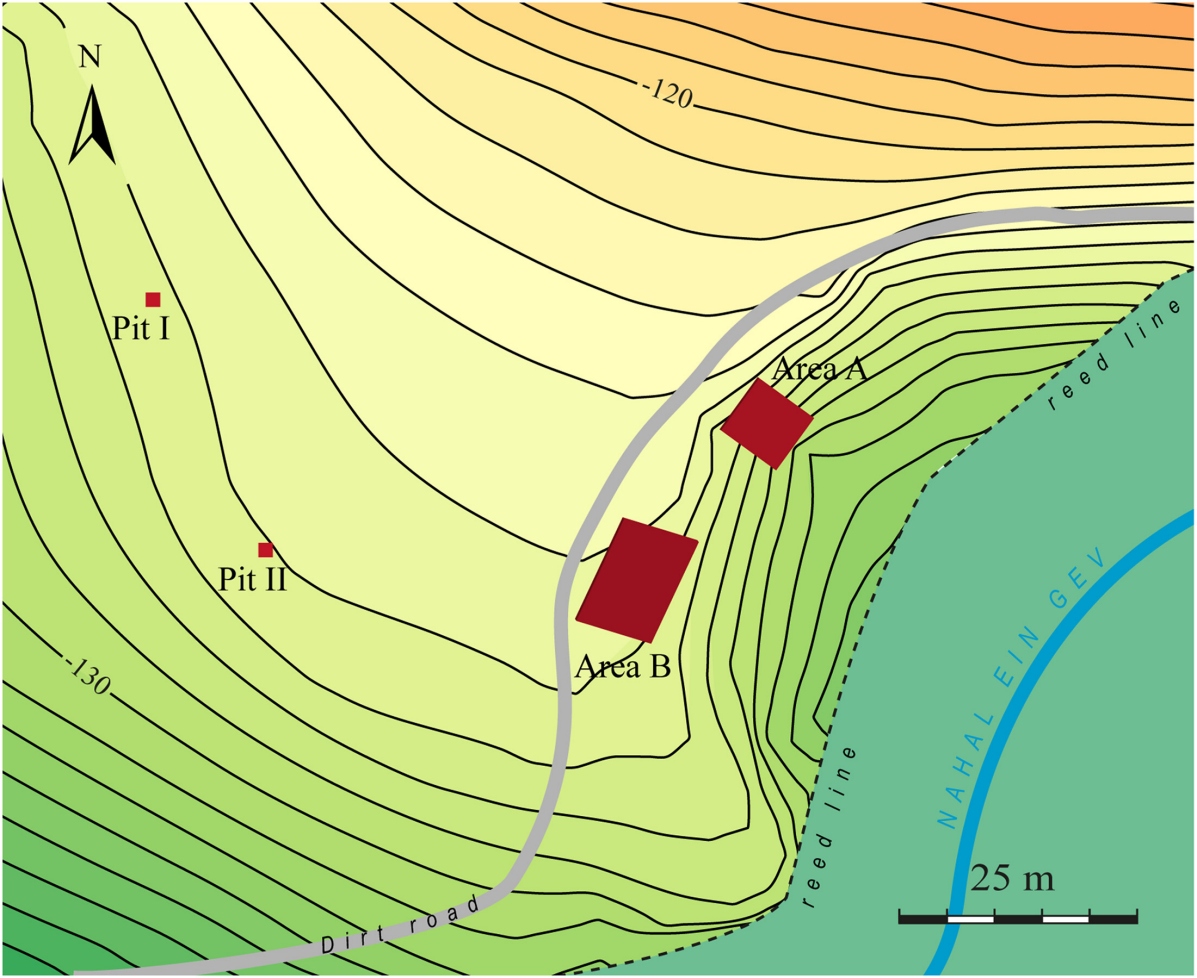
Plan of the site and the excavated areas.

Dense accumulations of lithics and architectural elements were distributed across the surface of the terrace close to the foxhole. Two test pits were excavated near the margins of the cultural accumulations on the surface to determine the size of the site (Fig 2). These pits yielded few material remains suggesting that the site extends between Areas A and B (see below) over an area of approximately 60x60 m (Fig 2). The site’s present surface was likely modified by historical agricultural activities. Excavations in Area A reached bedrock and indicate that the Natufians first settled on an uneven caliche surface (see below).

In the summer of 2010 excavations at the site were resumed for four consecutive seasons (2010–2013) (Israel Antiquities Authority Permits G-57/2010, G-49/2011, G-73/2012, G-76/2013).

The site was excavated in 50 x 50 cm units. Since no stratigraphy was visible in the cultural layer, units were excavated in 5 cm levels. Each locus and its associated features, unique artifacts, human remains and stones greater than 10 centimeters in length were mapped. All sediments were dry-sieved through 1.8 mm mesh. The sediment remaining in the screen was collected, wet-sieved and sorted for fauna and microartifacts such as microliths and beads. Soil samples were collected from every excavation unit. All soil samples, artifacts, human remains, and stones larger than 10 cm were recorded in the excavation digital database. During the first two seasons of excavation (2010–2011), one out of every three buckets of sediment was sampled for flotation. In 2013 only sediment from intact cultural contexts was floated. The bucket flotation yielded mostly very small fragments of unidentifiable wood charcoal. A few samples of identifiable plants were submitted for dating (see below). All other charcoal fragments recovered during the excavations were insufficient for dating. As in other Natufian sites in the region [12], the lack of seed remains prevented systematic botanical analysis.

Recovered materials (human burials, lithics, art, groundstone, bone tools) with the exception of the fauna are curated at the Institute of Archaeology, at the Hebrew University of Jerusalem. The fauna is stored in the Zooarchaeology Laboratory, in the Department of Ecology, Systematics and Evolution at the Hebrew University. To maximize our temporal and spatial understanding of the site we started digging in two areas along the eroded stream cut (*ca*. 54 m long) on the eastern edge of the site (Fig 2). Area A was opened to obtain a vertical section of the stratified occupation, while in Area B a large horizontal surface was exposed to investigate the spatial organization of the architectural features.

**Fig 3:**
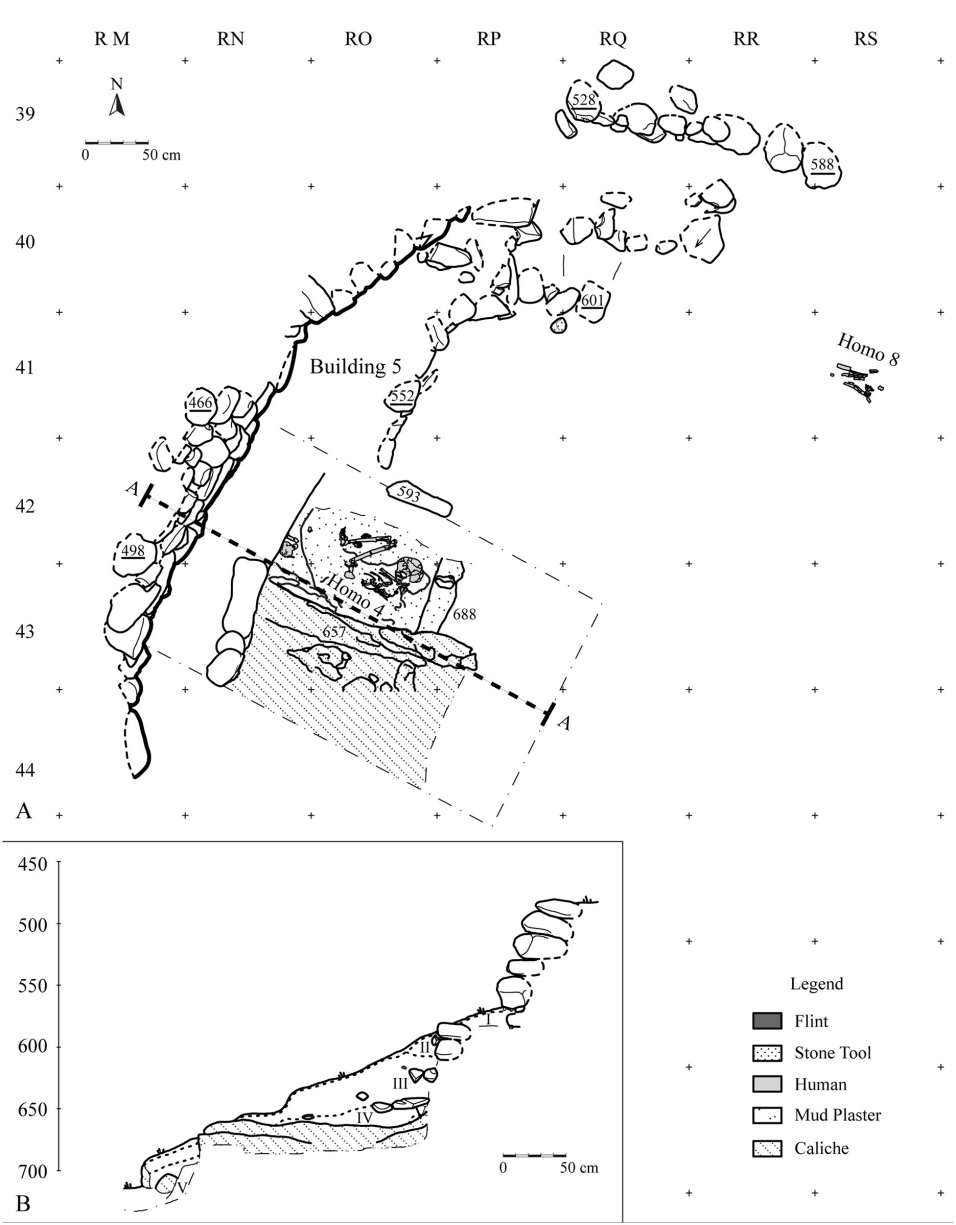
Area A: (A) plan and (B) section.

**Area A** (20 m^2^, 2 m deep) is located to the east of a 1 m high wall, initially observed in the erosional slope. The wall is built of uniformly sized, undressed stones and was initially thought to be a historical intrusion (Fig 3). Excavation revealed that the base of the wall continued on both sides of the original segment to form an oval-shaped structure facing the wadi (Fig 3). The base of the wall segment was embedded in Natufian sediments, negating its assumed historical origin. The excavation along the erosional slope was conducted as a series of steps exposing the entire stratigraphy of the site. From five cm below the surface, the deposits were homogeneous to a depth of *ca*. 2 m, suggesting a single occupation layer. Dense material remains, particularly lithic artifacts (see below), were distributed throughout the layer. At 2 m below the surface, solid caliche was exposed in several locations within the burial ground, while in other parts the dark gray sediments typical of the Natufian layer became mixed with fragments of lime plaster (Fig 3). To date human remains from at least four individuals have been retrieved from this area suggesting that a burial pit was dug into the caliche to inter several individuals (Fig 3).

**Fig 4:**
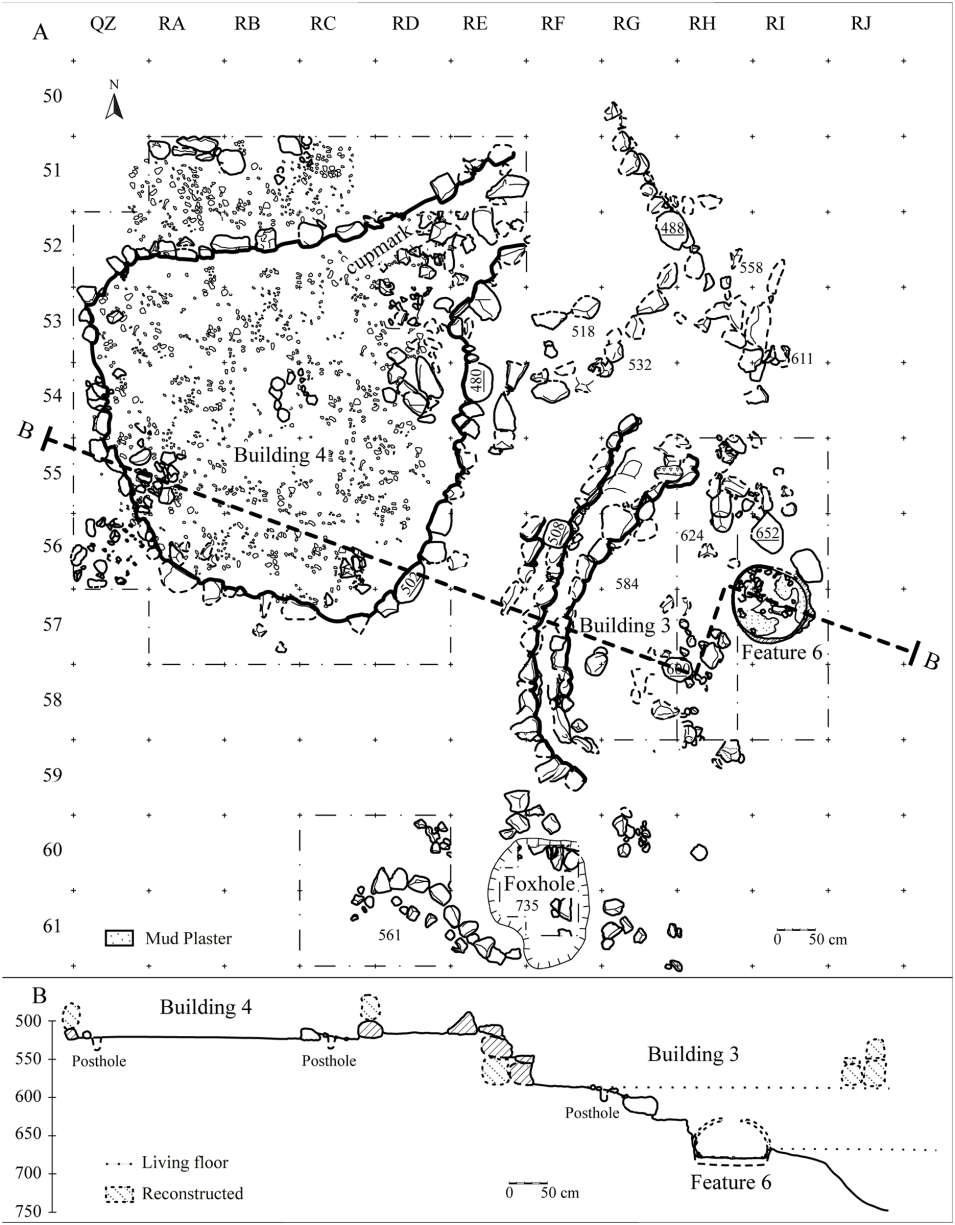
Area B: (A) plan and (B) section.

**Area B** (100 m^2^) is situated immediately to the west, north and east of the 1973 foxhole (Fig 4). In the eastern portion, the surface sediment was removed to a depth of 20 cm. The exposure of this large area revealed the bases of several stone structures and an installation (see below). The stratigraphy is quite uniform; the layer is gray, sandy, anthropogenic in nature and very rich in lithics, especially debitage, but also contains other cultural remains (e.g., shell beads, bones, etc.). In this area, the base of the site is about 2 m below the surface.

**Fig 5:**
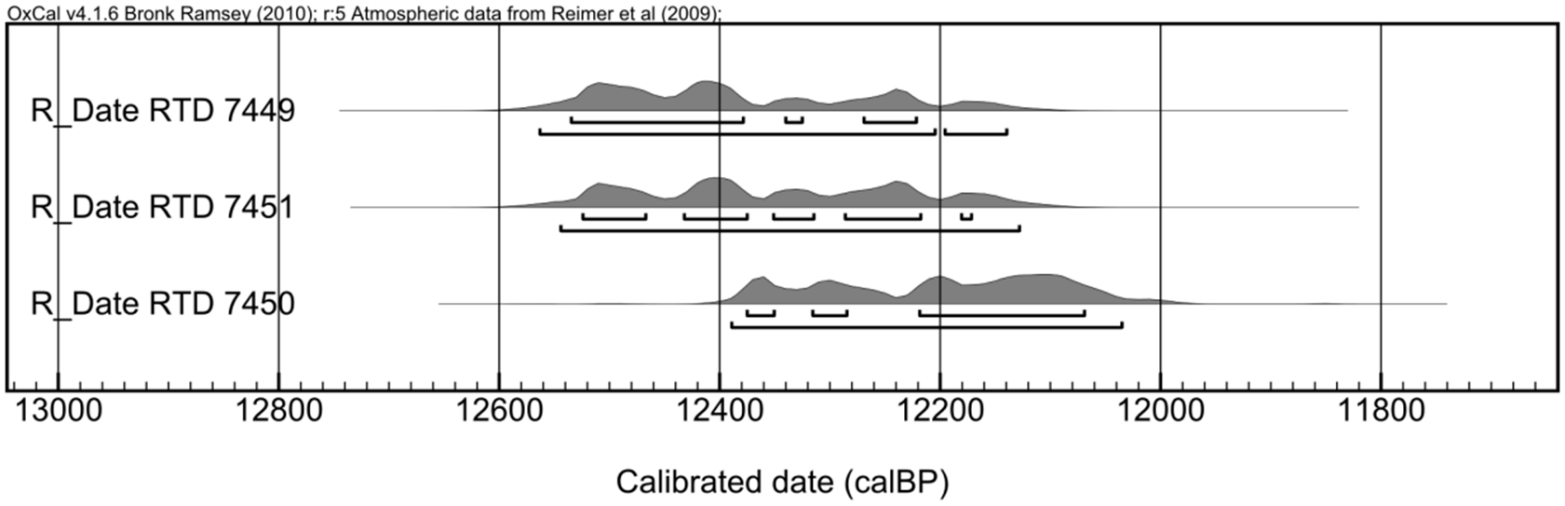
Probability distribution of the calibrated radiocarbon date ranges of the NEG II samples.

The pre-treatment of three charcoal samples, collected at about one meter below the Natufian surface, provided enough carbon for ^14^C dating. One sample derived from the top of the grave in Area A while the other two were collected from Area B close to the foxhole. The three samples were graphitized and measured by accelerator mass spectrometry at the D-REAMS radiocarbon dating laboratory at the Weizmann Institute of Science (Rehovot, Israel). The radiocarbon ages for the three samples cluster within 100 years of one another. Their calibrated ranges cover the period between 12,550 and 12,000 years cal BP (Table 1, Fig 5). This range falls within the Late Natufian chronology and is further supported by the lithic analysis (see below). It is interesting that samples RTD 1—Area A and RTD 2—Area B provide the same chronological date while RTD2 and RTD 3 which were recovered close together diverge by about 100 years.

**Table 1:** ^14^C ages are reported in conventional radiocarbon years (before present = 1950) in accordance with international convention [13]. All calculated ^14^C ages have been corrected for fractionation so that they are equivalent with the standard ^13^C value of -25‰ (wood). Following Reimer et al. (2013)[14]calibrated ages in calendar years were obtained from the calibration tables in OxCal v. 4.2 [15–17]. Laboratory number, field ID, type, species, pre-treatment efficiency (Eff%), carbon percent (C %), radiocarbon age and calibrated ranges in year BP for the ±1 standard deviation and ±2 standard deviation are provided for each sample.

Lab #	Field ID	Type	Species	Eff%	C%	14C age ±1σ year BP	Calibrated range ±1σ	Calibrated range BP ±2σ
**RTD-7449**	#10 RP42c	Charcoal	Quercus (it)haburensis	46.7	76.6	10460±45	12535 (55.4%) 12380	12550 (95.4%) 12135
							12325 (3.5%) 12310	
							12275 (9.3%) 12240	
**RTD-7451**	#16 RG60b	Charcoal	Tamarix	30.4	66	10445±45	12525 (16.3%) 12465	12540 (95.4%) 12120
							12435 (20.0%) 12375	
							12345 (27.9%) 12235	
							12200 (4.0%) 12180	
**RTD-7450**	#15 RG60d	Charcoal	Prunus Amigdalus	48.9	69.8	10340±40	12380 (11.1%) 12340	12390 (95.4%) 12000
							12300 (4.8%) 12285	
							12235 (52.2%) 12065	

### Architecture

**Fig 6:**
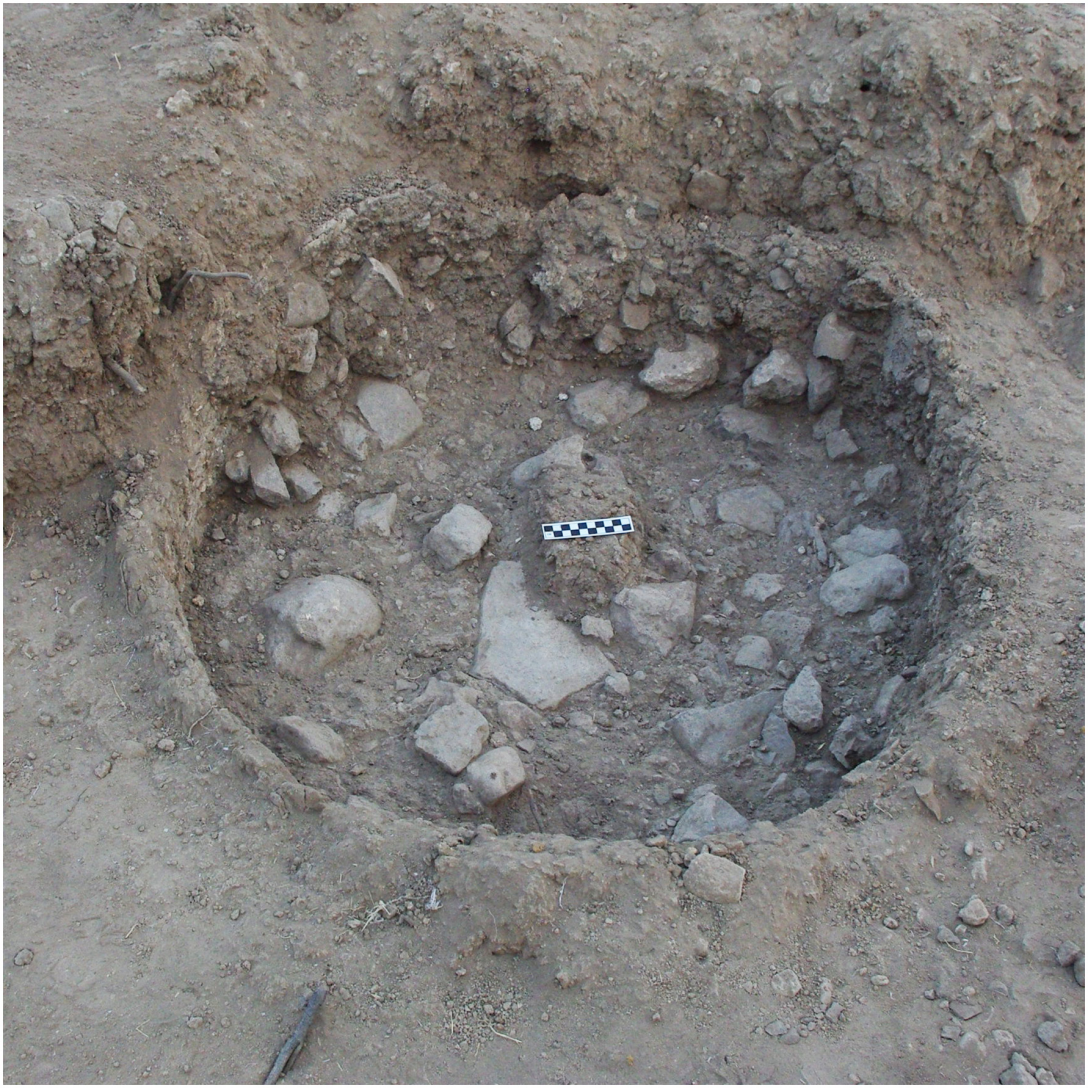
Rounded storage pit.

The thick Natufian occupation level at NEG II includes a series of architectural features, mostly massive rounded structures that reveal a complex site plan. The function of some architectural elements is not yet determined. These include a straight 5 m wall segment (Fig 4) and a rounded storage pit (80 cm in diameter at the base) that may have had a domed roof that collapsed (Fig 6).

Remains of many walls are visible in the eroded southeastern section. Partial walls of at least 8 structures built from undressed local basalt stones and limestone outcrops, were recorded. To better understand the architectural style of the Late Natufian at NEG II, three buildings (3, 4, 5) were excavated to their base:

#### Building 3 (Area B)

**Fig 7:**
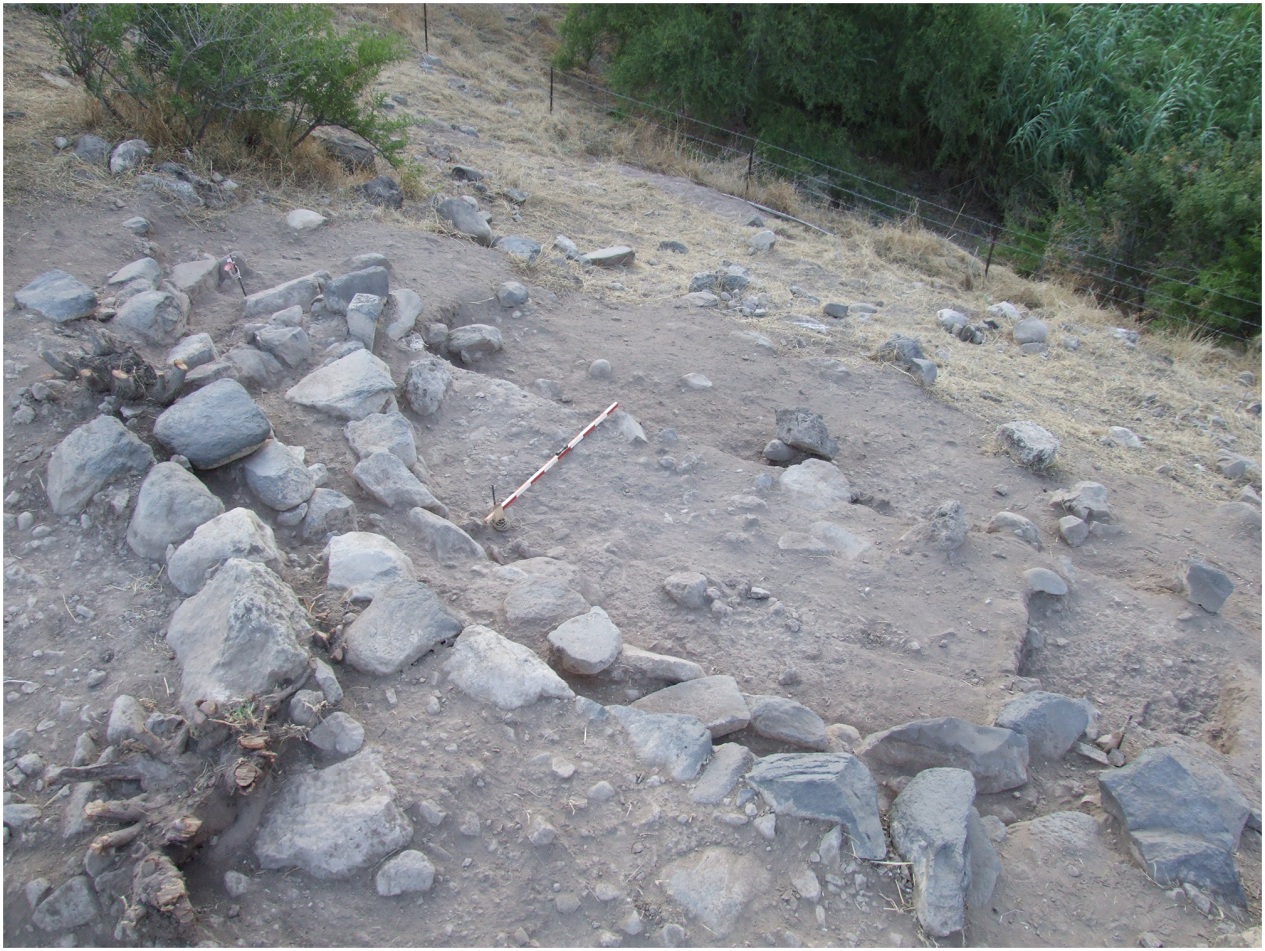
Building 3.

Building 3 is a large rounded structure cut by stream erosion. The curvature of the remaining walls suggests that the structure was about 5 m in diameter (Fig 7). The building is defined by an outer wall which is one course of stones higher than the attached inner wall. Several slabs were placed horizontally on the inner wall forming a bench. To better understand the base of this structure we excavated a section on its eastern side. The floor is indicated by a layer of small pebbles that underlie sediments adjacent to the base of the structure walls. The sediment below the surface is uniform and associated with three large stones that may be the remains of postholes penetrating the floor of the structure. The fill under the floor is gray, sandy, anthropogenic and rich in material remains, mainly lithics, but there were also bones, beads, etc.

#### Building 4

**Fig 8:**
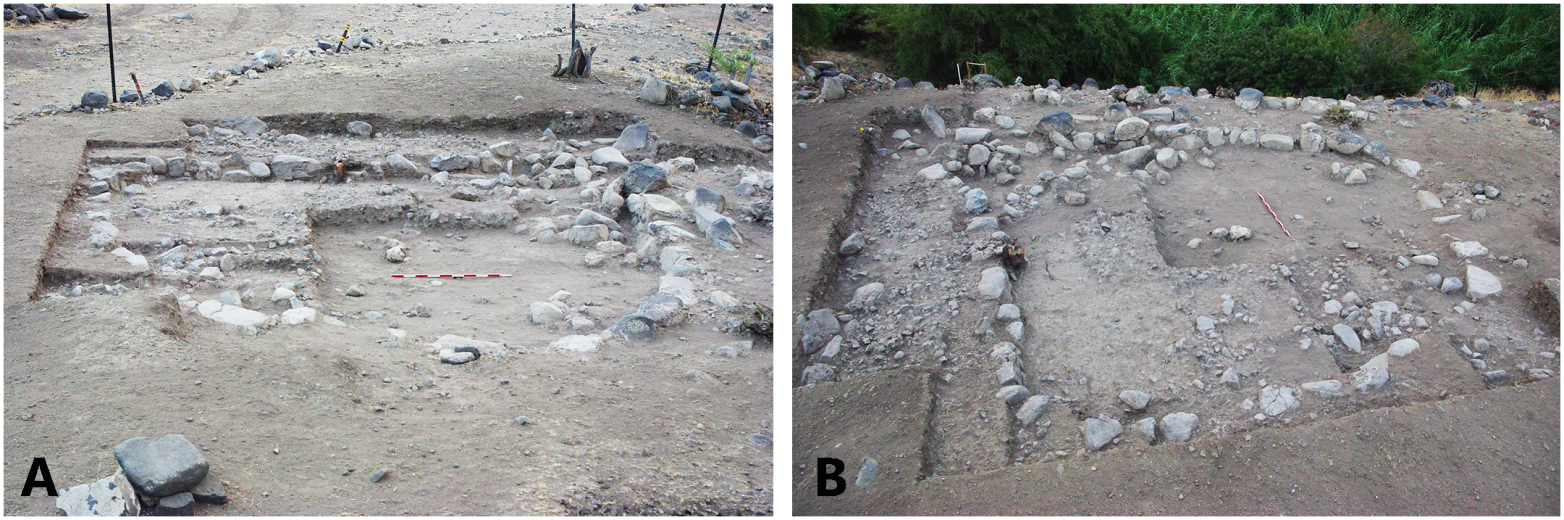
Building 4 –(A) view from the south and (B) from the west.

Excavation to the west of the foxhole revealed two aligned walls that curve in the opposite direction from the wall of Building 3 (Figs 4 and 8). We excavated along the inner wall until the complete contour of the structure was exposed. The shape of the structure is unusual: the southern end is oval, while the northern end is truncated by a straight wall (Fig 8). The stones that form the base of the wall are oriented with their flat side facing in. At the northwest corner two parallel walls connect to the structure. These walls are associated with a large tilted slab and several collapsed stones that may represent a specially arranged entrance. The first 15 cm of the sediments inside the structure were likely disturbed due to their proximity to the surface as indicated by the high concentration of lithic artifacts and the rarity of bone. However, the base of the structure at about 30 cm below the surface is characterized by compressed sediment and a high concentration of lithics and bones. Future excavations will verify whether postholes (represented by small piles of stones) and hearths (localities with ashes and burnt stones) were present in the structure. Several stones in the eastern wall were oriented horizontally and one is unique in size and heavily burned suggesting that it may have served as a hearth on the structure’s inner wall.

#### Building 5 (Area A)

**Fig 9:**
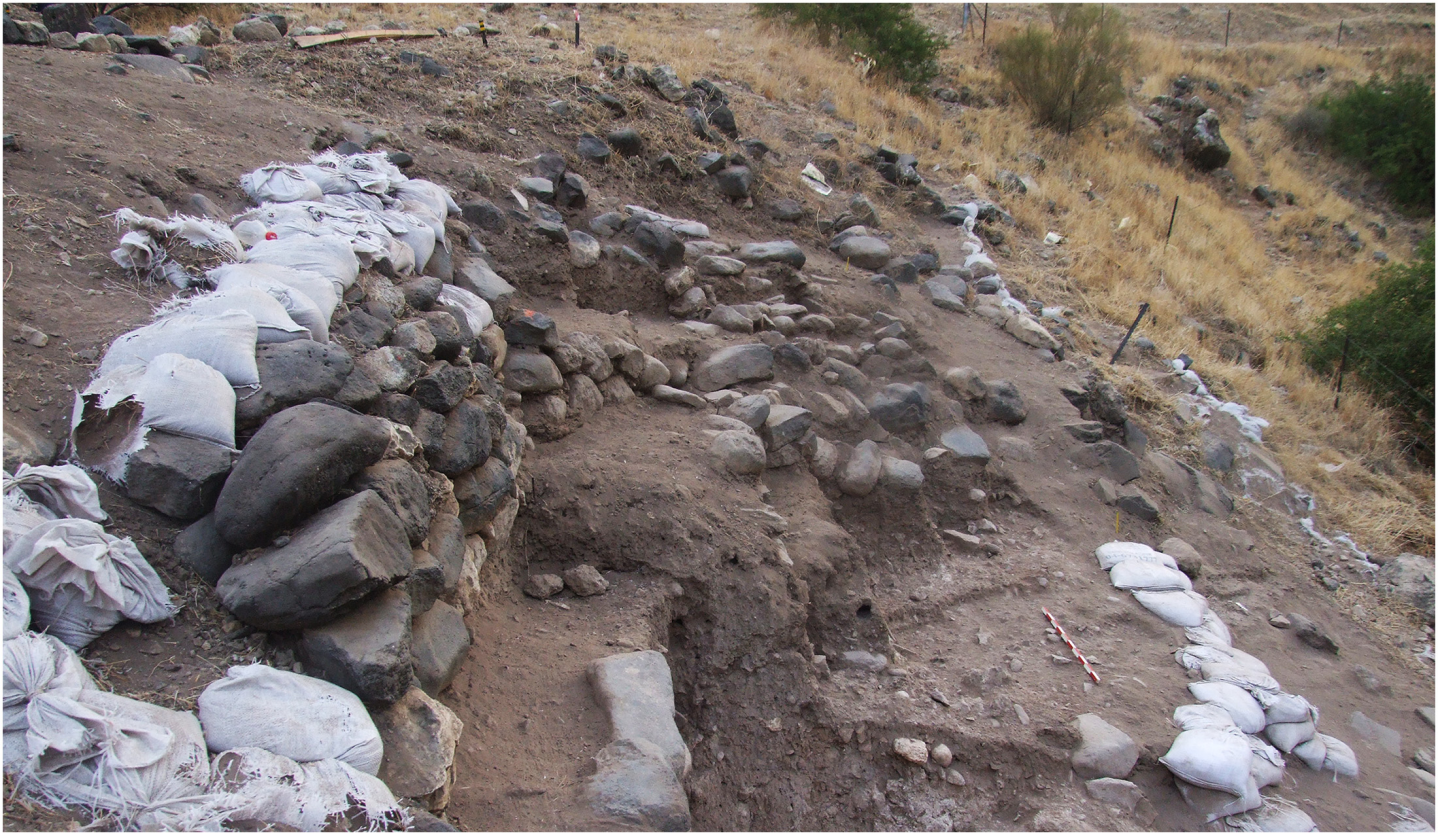
Building 5 –view from the south.

The wall of this structure is 90 cm high and is the best preserved wall excavated thus far. It is composed of 5 courses of limestone and basalt stones (Figs 3 and 9). The wall tilts slightly away from the structure to the northwest and two large stones mark its base at the top of the Natufian occupation level. Only the western side of the structure survived, yet the contour of the curve suggests a diameter of at least 5 m. A second wall was unearthed adjacent to the curved wall (Building 7), yet its stratigraphic association with Building 5 remains unclear.

In summary, our excavations reveal that the structures at NEG II were primarily large and rounded reaching about 5 m in diameter and in many cases were built with two parallel walls. The flattest surface of the top course of the stone blocks was oriented toward the inside of the structure. In two structures, horizontal stones were placed on top of the inner wall probably to serve as a bench, or hearth. Rich deposits of lithics and bones were distributed across the base of each structure often in powdery sediment. These carefully planned semi-subterranean houses were dug at least 30–40 cm into the ground and supported the superstructure of the dwellings. The foundations were made from undressed stones collected in the immediate vicinity of the site.

### Burials

Burials of several individuals have been retrieved from two locations on site.

**Fig 10:**
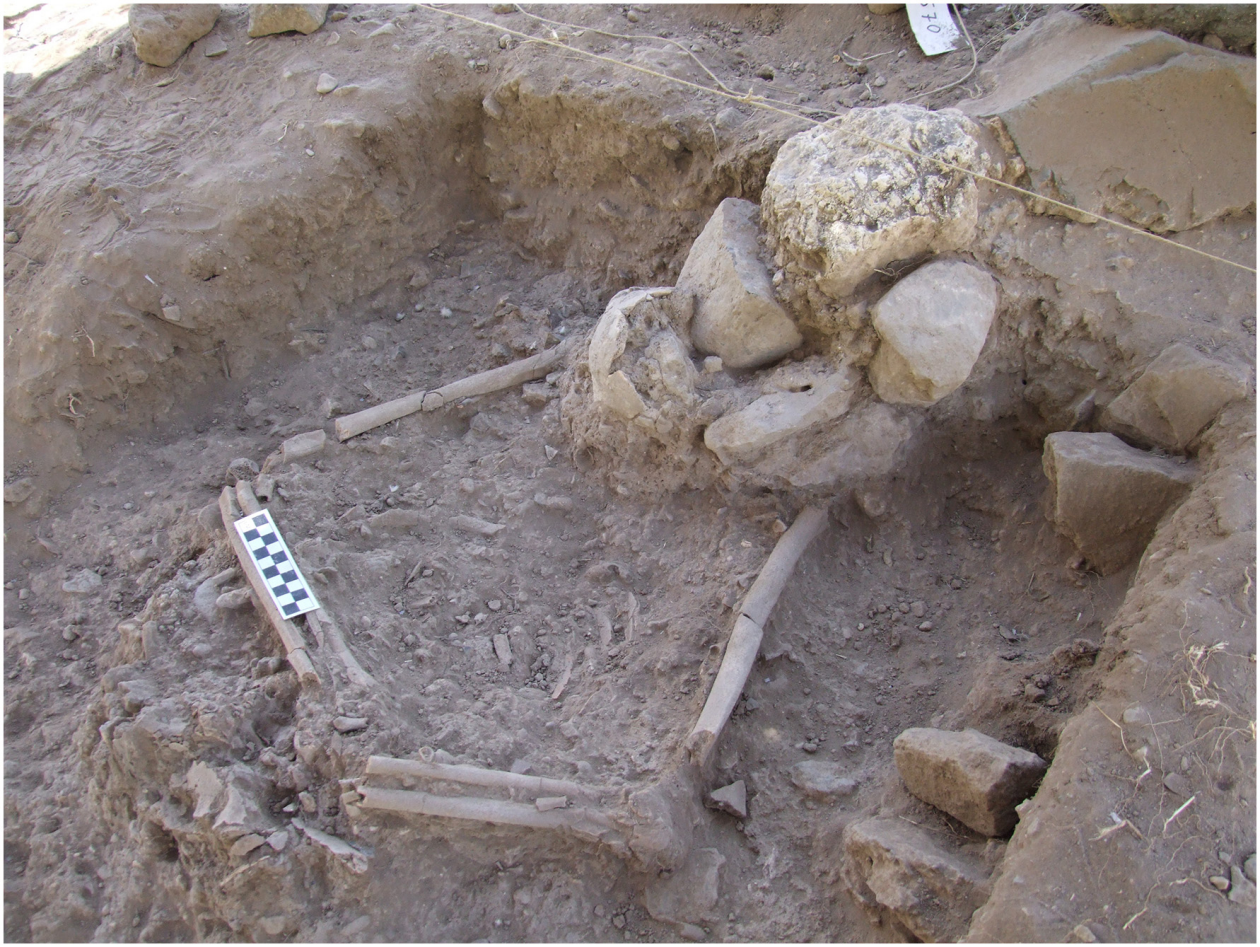
Homo 2.

**Area B**: a single primary burial of a female (Homo 2) was interred under the stones that form the southern wall of Building 3. The skeleton was embedded under the wall of a building that had likely been abandoned. The Natufians must have carefully chosen this location since they took apart the wall, interred the burial, and then reconstructed the wall which ultimately crushed the skull (Fig 10). The skeleton was positioned on her back in a tightly flexed position similar to the most common burials from the Final Natufian layer at Eynan [18]. The body was likely bound or wrapped with an organic material, perhaps a sack, but no clear edges of a burial pit are visible. The discrete but compact clustering of the bones indicates a clear boundary to the grave (see example [18]). The position of the body indicates that the wrappings or sack held the body in position as the joints disarticulated during decomposition. The tightly flexed legs were bent so that the knees were pressed to the upper chest with the arms wrapped around them placing the palms slightly above the pelvis. The head and neck were elevated slightly above the body during burial but rolled slightly to the side after disarticulation.

#### Area A—Multi-burial grave

**Fig 11:**
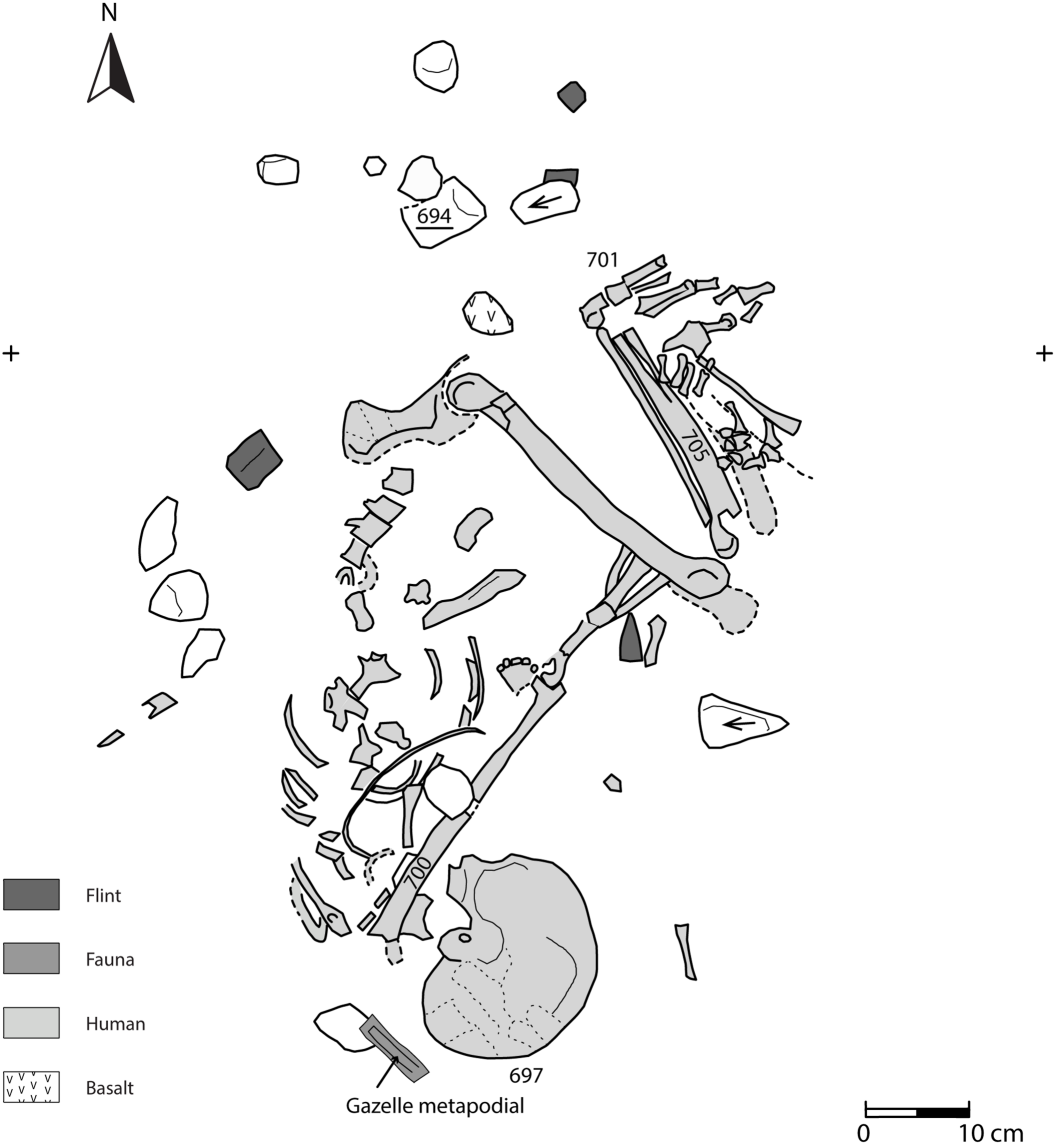
Homo 4—located at the base of the multi-burial grave.

Remains of at least four individuals were recovered from the burial pit in Area A described above. Stratigraphic observations suggest that the grave was used and then sealed prior to the subsequent Natufian occupation represented by 1 m of deposits above the grave (Building 5) (Fig 3). Recent excavations to the northeast indicate that the grave extends significantly beyond the currently exposed area, and will be investigated in future excavations. A female skeleton resting at the base of the pit (Homo 4, Fig 11) was laid on her right side in a completely articulated, flexed position. Her arms were placed parallel to one another between her legs, and her head was drawn toward the chest and faced downwards. The hard white sediment around her skeleton and the smoothed plaster under her skull suggest that her body was covered with lime plaster at the time of burial. The grave included bones of two other individuals (Homo 3, 5) that were scattered around Homo 4. This may indicate that Homo 4 was not the first individual to be buried in the grave and her skeleton disturbed at least two other previously buried individuals or that the bones of two later burials trickled into her grave after disarticulation.

The two complete burials recovered at NEG II were flexed—the dominant burial position in the Final Natufian occupation at Eynan [19]. No unique burial goods were recovered at NEG II, but special treatment of the dead is indicated by the lime plaster in Area A and the organic wrappings in Area B. The excavation of the large grave in Area A into the bedrock reinforces the suggestion that the burial ground served as the foundation for this Natufian occupation as evident in other Natufian sites [20, 21].

### The Lithic Industry

The detailed study of the lithic material from NEG II reveals similar techno-typological characteristics in the different contexts. Thus we have combined the lithic samples into one unified assemblage for the techno-typological study. The flint assemblage presented here comprises material from the 2010–2012 excavations of Areas A and B. The material from Area B includes the flint assemblages from Building 3, the grave of Homo 2 and the lowest 30 cm of the foxhole at the base of more than 2 m of anthropogenic sediments.

### Raw Materials

Most of the raw material derives from flint cobbles, probably from adjacent *wadis* and the shore of the Sea of Galilee. The colors of the flint are diverse. The most common are gray, brown, beige, reddish and purple. A black-bluish flint is of especially high quality, and unlike the *wadi* cobbles, has a calcareous cortex. Examination of the flint tools collected from adjacent Epipaleolithic sites in the Ein Gev region [22], less than 2 km southeast of NEG II, reveals the use of the same raw materials (including the black-bluish flint). There is evidence for the full *chaîne opératoire* of the production technology comprising primary elements, blanks, cores and tools. No preference for raw materials was observed for any particular tool category.

### Debitage

The debitage category (Table 2) comprises 8049 items. As the picking and sorting of the micro-components is not complete, we can only mention here that so far we have recovered 2.2 kg of chips and 1980 chunks. These items comprised the debris category encompassing artifacts smaller than 15 mm (chips) and those lacking signs of flaking (chunks).

**Table 2:** Debitage counts.

	Area A				Area B							
	Above 670*		Below 670*		Building 3		Homo 2		Foxhole		Total	
Debitage	N	%	N	%	N	%	N	%	N	%	N	%
Primary elements	666	19.3	235	19.7	428	26.1	146	19.6	250	24.9	1725	21.4
Flakes	2181	63.1	741	62.0	1063	64.7	479	64.4	550	54.7	5015	62.3
Blades	241	7.0	93	7.8	48	2.9	36	4.8	61	6.1	479	6.0
Bladelets	258	7.5	93	7.8	69	4.2	64	8.6	116	11.5	600	7.5
CTE**	95	2.8	29	2.4	33	2.0	16	2.2	26	2.6	201	2.5
BS**	14	0.4	5	0.4	1	0.1	3	0.4	2	0.2	25	0.3
MBT**	4	0.1	0	0.0	0	0.0	0	0.0	0	0.0	4	0.1
Total	3459	100.0	1196	100.0	1642	100.0	744	100.0	1005	100.0	8049	100.0
**Cores**												
One striking platform	40	50.6	8	50.0	6	50.0	2	50.0	3	42.9	59	50.0
Two striking platforms	14	17.7	0	0.0	1	8.3	0	0.0	3	42.9	18	15.3
Bidirectional striking platforms	7	8.9	3	18.8	1	8.3	1	25.0	0	0.0	12	10.2
Three striking platforms	2	2.5	1	6.3	0	0.0	1	25.0	0	0.0	4	3.4
Others	16	20.3	4	25.0	4	33.3	0	0.0	1	14.3	25	21.2
Total	79	100.0	16	100.0	12	100.0	4	100.0	7	100.0	118	100.0
Debitage	3459	87.0	1196	82.8	1642	94.2	744	93.6	1005	90.0	8049	88.7
Cores	79	2.0	16	1.1	12	0.7	4	0.5	7	0.6	118	1.3
Tools	436	11.0	232	16.1	89	5.1	47	5.9	105	9.4	909	10.0
Total	3974	100.0	1444	100.0	1743	100.0	795	100.0	1117	100.0	9076	100.0

* Below datum

**CTE = core trimming elements, BS = burins spalls, MBT = microburin technique.

The debitage is dominated by flakes, as is typical of Natufian and PPNA assemblages in the region [23–26]. The ratio of flakes:blade/lets is 4.6:1 while the ratio of blade:bladelet is 1:1.2. The relatively equal ratio of blades and bladelets is more typical of PPNA than Epipaleolithic assemblages, although there are some exceptions [27]. Blank preference for tool manufacture is the opposite, *i*.*e*., there is an obvious bias towards blade/lets blanks. The ratio of tools on blade/let:flake blanks is 3.8:1 and the ratio of blade:bladelets is 1:1.3.

Primary elements (with at least 25% cortex on the dorsal surface) comprise 19% of the debitage. This contrasts with the very low ratio of cores:debitage (1:62.2) and the high ratio of tools:debitage (1:8.8). It may be related to the fact that most of the flint assemblage derives from dwellings and graves and we have not yet discovered a knapping area.

**Fig 12:**
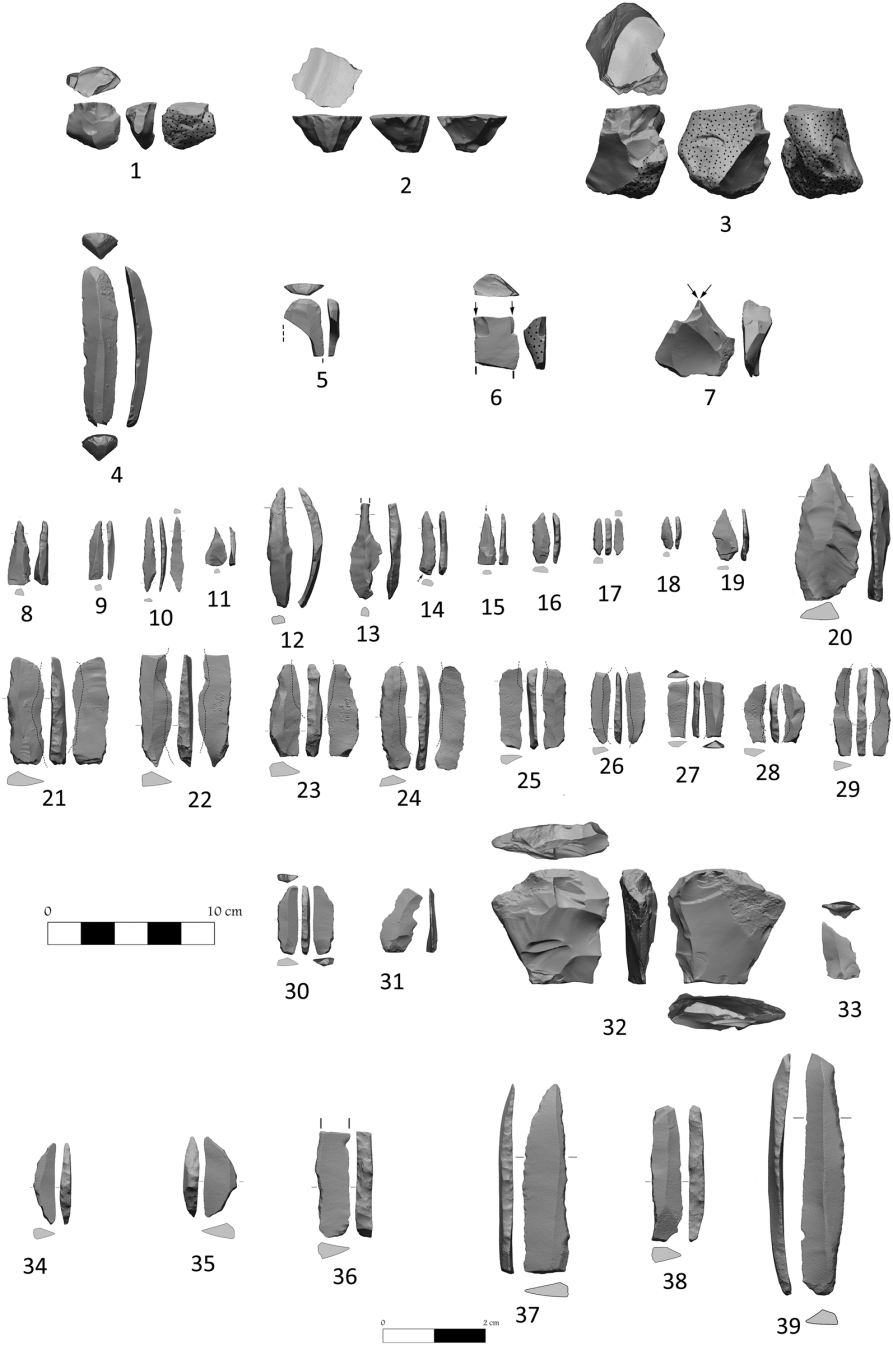
NEG tools: 1–3: cores; 4–5 scrapers; 6–7: burins; 8–20: perforators; 21–29: sickle blades; 30: backed blade; 31: notched; 32: varia; 33: truncated; 34–35:lunates; 36–39:backed bladelets.

The tendency to produce flakes is also observed on the cores. Of the 188 cores, more than half (53%) display scars of flake removals, while only 16% display blade/let removals (Fig 12:1–3). The rest of the cores have scar patterns of both flakes and blade/lets. Half of the cores have one striking platform and *ca*. 25% of the cores have two striking platforms, most are not opposed (Table 2). The rest of the cores include items with three striking platforms, radial and amorphous cores and core fragments. The majority (66%) of the cores have flat striking platforms. The striking platforms of the remaining cores were shaped by one to three small flake removals. Nearly 85% of the cores display hinged fractures that terminated the use of the core. In many Late Epipaleolithic assemblages the cores are discarded once a hinge fracture prevents further use, but this usually occurs after they have reached a small size. At NEG II the discarded cores are relatively large and in many cases the hinged cores may have been repaired. This *ad hoc* character of the cores is also reflected in the core trimming element (CTE) subtypes, which include only a few ridge blades and core tablets. It is also worth noting that although the NEG II inhabitants were familiar with the microburin technique (MBT), they did not use it systematically. Only four MBT items were recovered and only five microliths bear the typical scar left by this technique.

### Tools

There are 909 tools in the assemblage (Table 3). The main tool categories include perforators, microliths, and retouched and backed items (Fig 12). The rest of the toolkit contains burins, notches/denticulates, truncations and endscrapers as well as items assigned to the *varia* and composite tool categories. Not a single item in the current assemblage can be considered a *fossile directeur* of the PPNA (e.g. Khiam and/or Salibiya points, Beit Ta’amir sickle blades, Hagdud truncations, tranchet axes, etc.).

**Table 3:** Frequencies of the tool categories.

	Area A				Area B							
	Above height 670*		Below height 670*		Building 3		Homo 2		Foxhole		Total	
Tools	%	N	%	N	%	N	%	N	%	N	%	N
Endscrapers	3.0	13	0.9	2	4.5	4	8.5	4	4.8	5	3.1	28
Burins	4.6	20	3.0	7	3.4	3	4.3	2	8.6	9	4.5	41
Perforators	35.6	155	41.4	96	30.3	27	12.8	6	24.8	26	34.1	310
Backed Items	9.4	41	4.3	10	18.0	16	17.0	8	9.5	10	9.4	85
Truncations	2.5	11	5.2	12	4.5	4	2.1	1	3.8	4	3.5	32
Notches and Denticulates	4.1	18	3.0	7	4.5	4	2.1	1	4.8	5	3.9	35
Retouched Items	15.4	67	12.9	30	13.5	12	17.0	8	8.6	9	13.9	126
Composites	0.5	2	1.3	3	0.0	0	2.1	1	0.0	0	0.7	6
Varia	6.4	28	3.0	7	4.5	4	4.3	2	5.7	6	5.2	47
Non-Geometric Microliths	14.5	63	20.3	47	13.5	12	19.2	9	22.9	24	17.1	155
Geometric Microliths	4.1	18	4.7	11	3.4	3	10.6	5	6.7	7	4.8	44
TOTAL	100.0	436	100.0	232	100.0	89	100.0	47	100.0	105	100.0	909

* Below datum.

The perforators are the most common tool type (*ca*. 34%, Fig 12:8–20). They are usually found in low quantities at Natufian sites, but are very common in the PPNA [28]. Because they are heterogeneous in nature, it is very difficult to ‘draw a line’ between borers and awls according to Bar-Yosef's type-list [22]. Still it is important to emphasize that ‘classic’ awls (items with two notches and a pointed tip) that are very common in PPNA sites such as Netiv Hagdud [26] are rare in the NEG II assemblage. In general the perforators of NEG II fit better with Bar-Yosef's definition of borers or *mèche de foret* according to Tixier type-list [29]. Approximately 50% of the perforators have microlithic dimensions. Because they were formed by abrupt retouch on both lateral edges, it is impossible to be certain of the original size of the blanks, yet the perforators usually have the dimensions of bladelets and blades. Items with one working edge are most common, but items with both distal and proximal edges are also represented (*ca*. 14%, excluding the broken items).

The microlithic tools are an important component of the assemblage (non-geometric—*ca*.17%; geometric -*ca*.5%). The dominant non-geometric microliths are backed bladelets, mostly with a straight back. Fine and semi-abrupt retouch mostly on the dorsal edge is present on some bladelets, as is inverse retouch. The geometric items comprise 38 lunates and 4 triangles. All lunates are backed, either by unipolar (60.5%) or bipolar retouch (39.5%)—an anvil was likely used for the latter. The average dimensions of the lunates are 13.3 mm in length (N = 29), 4.7 mm in width (N = 38) and 2.2 mm in thickness (N = 38). The size and shape of the lunates are similar to those reported from other Late Natufian [30, 31]and Harifian assemblages from the Negev [32]. Only one lunate has a typical MBT scar, but MBT scars are often obliterated by reshaping and retouch. Interestingly, this assemblage does not contain even one Helwan retouched microlith. The retouched pieces are the third largest tool category in the assemblage (*ca*. 14%). Most are retouched blades (*ca*. 59%, not including fragments).

Another important category is the backed items. It comprises *ca*. 9% of all the tools and is dominated by sickle blades (*ca*. 35%) and backed blades (*ca*. 31%). Sickle blades are one of the few standardized tool types in this assemblage. The average dimensions of the sickle blades are quite large: 42 mm in length (N = 7), 13 mm in width (N = 29) and 5 mm in thickness (N = 30). Since only seven sickle blades are complete, it is hard to define their shape beyond the abrupt and bipolar lateral backing and their thickness. The most common backed blades have straight backs, while others have a curved or concave back. Most were shaped by abrupt retouch, but some were modified by bipolar backing.

The rest of the tools belong to the following categories: burins (*ca*. 4%), most of them dihedral on natural surfaces; notches/denticulates (*ca*.4%), usually made on flakes; straight and oblique truncations (*ca*. 3%); endscrapers (*ca*.3%), almost all on flakes; and composites (*ca*. 1%), all either endscrapers or burins on one edge and another tool type on the other. Almost 5% of the tools were assigned to the *varia* category. Of interest are items (N = 28; 59.7%) whose rather shallow flaking on the ventral face precludes them from being categorized as cores-on-flakes (Fig 12:32). These also lack an acute working edge, and thus could not be defined as flat burins. Neither do they fall within the definition of *pièces esquillées* (only three items are flaked bidirectionally, the rest are unidirectional) or Nahr Ibrahim truncations (only a few of them have a prepared surface for the removals). These were most likely used as hammer stones or *ad hoc* tools for a specific, yet unknown purpose. Three other items in this category are heavy-duty tools; two are made on thick cortical flakes and the third was manufactured from a nodule. One item is a bifacial tool that lacks a tranchet blow. The rest of the *varia* are items that do not fit into any of the other tool categories.

The technological characteristics of the assemblage are typical for Levantine Late Epipaleolithic entities. To obtain a desired tool shape, the knappers focused more on retouch and less on producing pre-shaped, standardized blanks [33, 34]. This is reflected in the different ratios of flakes:blades/bladelets in the debitage (4.6:1) than the tools (1:3.8). The minimal investment in core preparation is evidenced through the rarity of core tablets and ridge blades, the cursory exploitation of the core volume [25] and the preference for abrupt and bipolar retouch to modify the blanks into tools (62.1%).

Three tool categories each comprise more than 10% of the total tool count. The largest category is the perforators that comprise more than a third of the tools. The second is the non-geometric microliths and the third is the retouched pieces. In the publication of the material from the test pit dug in 1973 [11] it was noted that in contrast to findings from other Late Epipaleolithic and PPNA sites in the region borers outnumber awls in the perforators category. This conclusion is still valid for the current excavations, as is the fact that classic awls are quite rare in both assemblages.

The sickle blades are also of interest. These are similar to those reported from other Late Epipaleolithic assemblages and are more backed than PPNA examples. It is worth noting that although the microliths comprise quite a big part of the assemblage, they are less common than in other Late Epipaleolithic sites in the region [28], although the size and shape of the lunates are similar [30–32]. No typical PPNA artifacts were identified in the material from the 2010–2012 excavations.

### The Faunal Assemblage

The faunal assemblage from NEG II reflects intensive exploitation of habitats in the Jordan Valley and the Lower Golan. Diversity and relative taxonomic indices, surface damage, and gazelle mortality profiles reveal an assemblage formed from a range of animal butchery and consumption events, the hunting of a broad spectrum of taxa including many low-ranked types and a high proportion of yearling gazelles.

Analysis of the NEG II faunal assemblage is ongoing—this report includes only the identifiable material recovered during the 2011 and 2012 excavation seasons (NISP = 2240 identifiable specimens) from all excavation areas. A high proportion of the faunal specimens from NEG II are identifiable to element and taxonomic group (ca. 25% of total fauna). Nevertheless, the fauna is significantly less abundant by volume than the lithic assemblage. This is at least partially related to conditions of preservation especially in the near surface layers. Faunal preservation at the site improves as depth from the surface increases.

The NEG II assemblage is clearly anthropogenic—fractures are typified by fresh spiral breaks (88.4%), cutmarks (0.6%) and burning (8.8%) and are distributed over a wide range of body-parts attesting to human butchery and in some cases cooking. Damage from natural processes such as animal activity and weathering is rare.

### Relative Taxonomic Abundance

**Fig 13:**
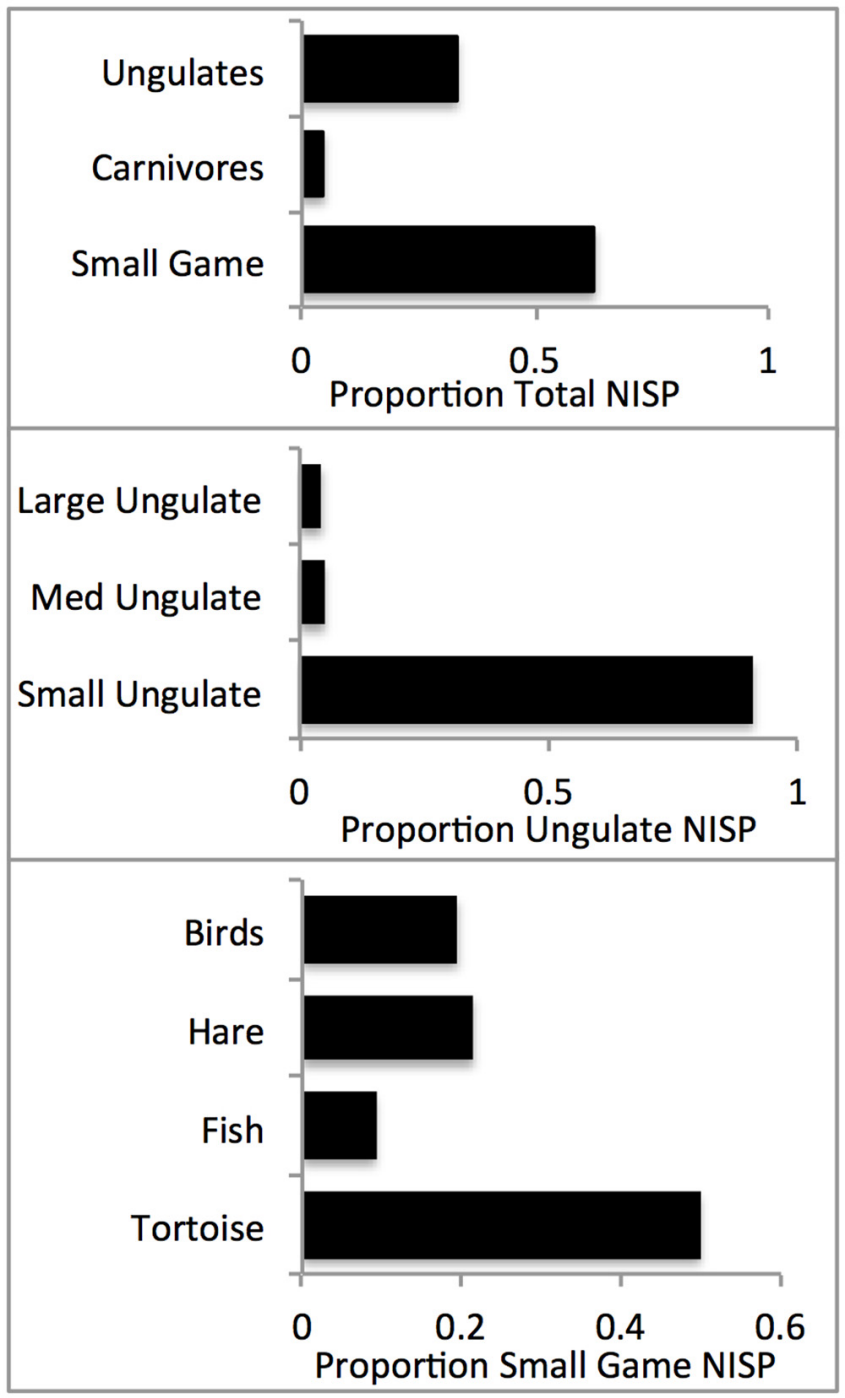
Relative abundance indices including broad taxonomic groups, ungulates and small game taxa.

In most respects, the faunal spectrum encompasses the range of taxa typical of the Natufian culture including ungulate (33%), carnivores (4%) and small game (63%) (Table 4). The assemblage can be distinguished from those of other Natufian sites in its abundance of fish and waterfowl that appears to be related to the site’s environmental setting in a riparian habitat close to the Sea of Galilee. The ungulate fauna is abundant and rich (n = 699; n taxa = 8; Table 4), but very uneven (Simpson’s Index of Diversity = 0.02), as specimens assigned to species are nearly entirely gazelle (91%; n = 434). Most of the less diagnostic fragments assigned to the small ungulate category (n = 231) are likely also gazelle and are thus combined with the gazelle (*Gazella gazelle*) in the remaining analyses. Red deer (*Cervus elaphus*), fallow deer (*Dama mesopotamica*), roe deer (*Capreolus capreolus*), wild boar (*Sus scrofa*), wild goat (*Capra aegagrus*), wild cattle (*Bos primigenius*) and wild ass (*Equus hydruntinus*) are all present in the assemblage in very small numbers (<10 NISP each) (Fig 13).

**Table 4:** NISP of taxa represented at NEG II.

LATIN NAME	COMMON NAME	NISP
UNGULATES		
*Equus sp*.	Wild ass	2
Cervidae	Indet cervid	4
*Capreolus capreolus*	Roe deer	10
*Dama mesopotamica*	Fallow deer	5
*Cervus elaphus*	Red deer	4
*Bos primigenius*	Wild cattle	7
*Sus scrofa*	Wild boar	5
*Capra aegagrus*	Wild goat	1
*Gazella gazella*	Mountain gazelle	396
Small Ungulate	Indet	231
Medium Ungulate	Indet	20
Large Ungulate	Indet	12
Huge Ungulate	Indet	2
UNGULATE TOTAL		699
CARNIVORES		
*Vulpes vulpes*	Red fox	72
Canidae	Indet Canid	2
*Felis silvestris*	Wildcat	9
Large Felid	Indet	1
*Martes foina*	Stone/Pine marten	3
*Meles meles*	Badger	1
Mustelidae	Mustelid	1
Small Carnivore	Indet	5
CARNIVORE TOTAL		94
SMALL GAME		
*Potamon potamios*	Crab	9
*Testudo graeca*	Med Spur-thighed Tortoise	663
Snakes	Indet	100
Lizard	Indet	1
Pices	Indet Fish	23
Cyprinidae	*Barbus/Capoeta* sp.	99
Falconiformes	Indet.	7
*Aquila chrysaetos*	Golden eagle	2
*Buteo buteo*	Buzzard	1
*Aegypius monachus*	Cinereous vulture	1
*Alectoris chukar*	Chukar partridge	51
*Anser* sp.	Goose	39
*Anas platyrhynchos*	Mallard duck	1
Columbiformes	Dove	1
Small Bird	Indet	6
Medium Bird	Indet	67
Large Bird	Indet	77
Huge Bird	Indet	12
*Erinaceus europaeus*	European Hedgehog	2
*Lepus europaeus*	European hare	285
SMALL GAME TOTAL		1447
GRAND TOTAL		2240

The carnivore assemblage is also typical in its abundance and composition. Red fox (*Vulpes vulpes*) is the most common taxa (82.8% of carnivores), followed by wild cat (*Felis silvestris*; 9.6% of carnivores) and a couple of Mustelids (*Martes foina* and *Meles meles*; 5.3% of carnivores). In sheer numbers, however, the best represented prey in the NEG II assemblage are small game types (63.3%, Fig 13). The usual Natufian species are abundant (tortoise [*Testudo graeca*] - 50%; hare [*Lepus capensis*]- 21%; and chukar partridge [*Alectoris chukar*] - 3.8%) but geese (*Anser* sp.; 8.7%) and fish (9.1%), nearly exclusively large barbels (*Barbus* and *Capoeta* sp.), also figure prominently (Fig 13).

### Gazelle Mortality

**Fig 14:**
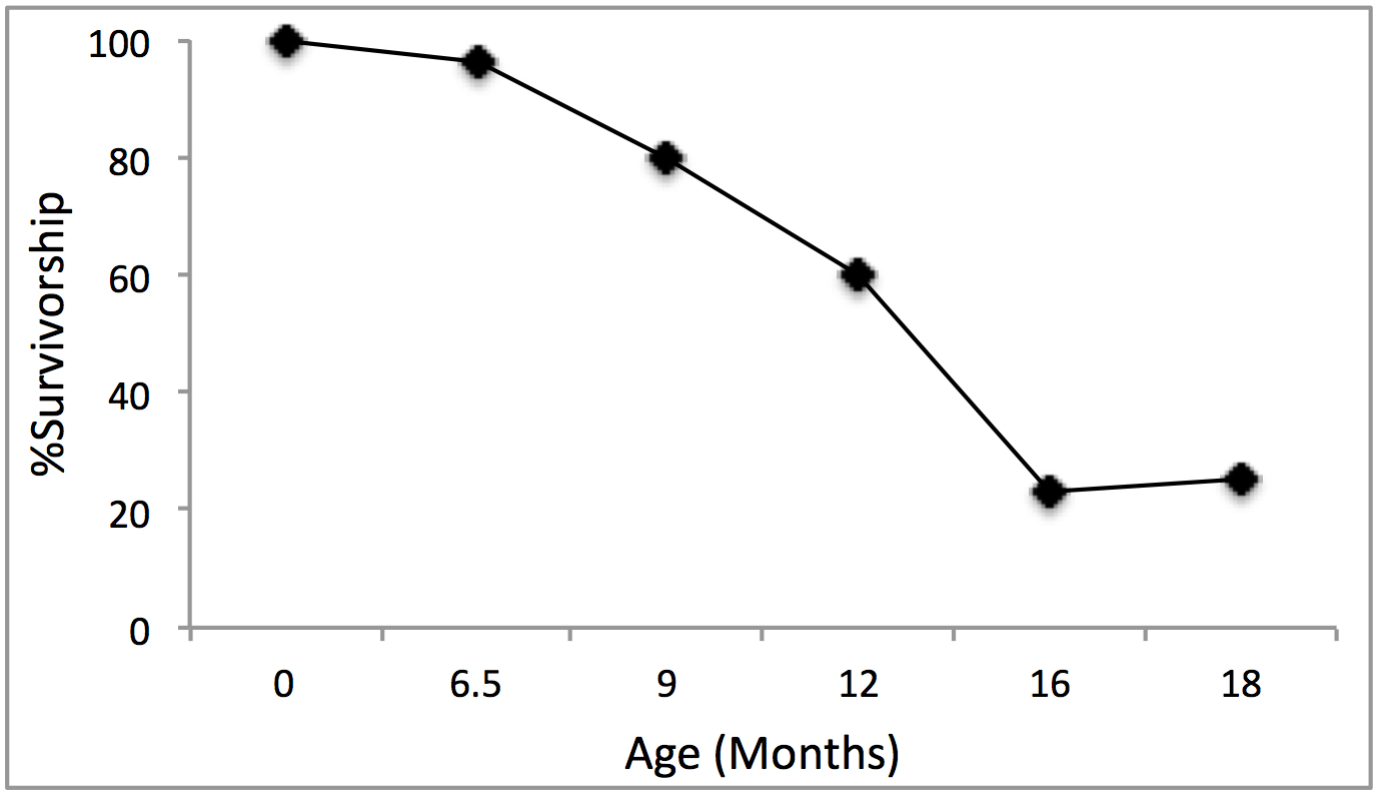
Survivorship curve of gazelles from NEG II based on the proportion of fused first and second phalanx (6.5 mos), distal tibia (9 mos), tuber calcis of calcaneum (12 mos), distal metapodial (16 mos), distal radius (18 mos) [35,36].

Unfortunately, the gazelle tooth sample is too small to be informative. Postcranial bones are much better represented and the high proportion of fused first and second phalanges (96.2%; MNE) confirms that very young gazelle (fetuses, newborns and fawns less than 6 months of age; [35, 36]) are rare. Only one specimen derives from a fetus or neonate. Nevertheless, the survivorship curve constructed from elements that fuse at different ages (Fig 14) reveals a steep drop-off in survivorship after about one year of age. Seventy-seven percent of distal metapodials (MNE = 13) are unfused, indicating that less than one quarter of the hunted animals were older than 18 months of age. Gazelles reach full-body size and sexual maturity at 18 months, so yearling animals are nearly full grown. Although the residents of NEG II hunted few fawns, they hunted yearlings in proportions far greater than expected for a stable population (23–46% depending on the season; [37]).

### Zooarchaeology Overview

The wide range of taxa, the abundance of small game, the wide range of habitats exploited and the focus on young gazelles reflect an intensive hunting strategy at NEG II especially in comparison to earlier Epipaleolithic and Paleolithic assemblages in the region, and perhaps more importantly to many other Late Natufian assemblages in the southern Levant [36, 38–43]. The focus on lower-ranked taxa enabled the Natufians to effectively combat resource stress on two fronts—first, by increasing the amount of animal products that they could extract from a given area and second, by buffering themselves against further stress. Despite high capture costs, these new taxa are significantly more abundant and demographically resistant to intensive hunting than the higher-ranked staple taxa of the Paleolithic (larger ungulates and tortoises). The abundance of aquatic small game at NEG II differs from sites in the Mediterranean zone where fish and waterfowl were not as readily accessible. Fish from sites in the Mediterranean region mainly include tiny freshwater types that likely originated in local riverine systems, with occasional specimens from the Mediterranean sea (i.e., sea breams) [44]. The fact that the Natufians were able to adapt their diverse exploitation strategies to capture a variety of small game taxa in a complex habitat requiring special technologies (e.g., nets, traps, hooks, barbs, harpoons) attests to their flexibility as hunters as well as their continued need to expend significant energy in the capture of less cost effective animals.

The abundance of yearling gazelles in the assemblage supports a pattern of intensive hunting in two dimensions. First, the living population of gazelles available to the Natufians was younger on average than a stable gazelle population indicating a high rate of population turnover characteristic of a growing population (mortality exceeds fertility). In growing populations lifespan is shorter, juvenile mortality declines and more young may be produced [45]. Second, the hunters from NEG II broadened the range of their preferred prey, taking not only the highest-ranked, adult gazelles as they did in the Paleolithic and Early Epipaleolithic, but the lower-ranked smaller juveniles as well. As in their addition of low-ranked small game animals, broadening their diet to include younger age groups, enabled them to extract more meat out of the surrounding area. Nevertheless, they stopped short of including gazelle fawns in their hunting repertoire.

### Art

A rich art assemblage including personal ornaments, modeled limestone items and engraved bone and stone objects was retrieved from the 2010–2013 excavation seasons at NEG II. Of the 210 personal ornaments recovered from the site, the majority are beads (n = 160), mainly disc- or cylindrical-shaped (n = 50).

Most of the disc beads are made from at least two species of Cardium shells (*Cerastoderma glaucum* and *Acanthocardia* sp.). While both are Mediterranean species, a geological outcrop containing *Acanthocardia sp*. fossils was discovered 100 m northeast of the site attesting to the use of both local and imported Mediterranean shells as raw materials. Evidence for on-site production of Cardium disc beads is manifested in the presence of items in various stages of completion. These are used to reconstruct the process of manufacture. Both the production sequence (*chaîne opératoire*) and the final product were standardized. Preforms and unfinished items share similar attributes and reveal a three step production sequence: (1) cutting a circular polygon (ca. 8 mm in diameter) from a shell valve; (2) drilling a conical hole through the center of the polygon from the inner face of the shell valve; and (3) polishing the bead to obtain a circular form and a smooth surface by removing the higher ribs of the shell. The diameter of the beads was not constrained by the size of the raw material but ranges between 3–12 mm (average 7±2.3mm), which is smaller than the valve of the shell. The Cardium disc beads manufactured at NEG II reflect a Late/Final Natufian innovation in shell bead manufacture, a new form that is not restricted by the original shape of the shell [46].

The form and technology of the NEG II disc bead assemblage are shared by contemporaneous Final Natufian sites in the Jordan Valley, Eynan and Huruq Musa [19, 47–49]. At NEG II Cardium disc beads were manufactured from local Cardium fossils and imported Mediterranean shells, while at Eynan and Huzuq Musa they were made only from imported, Mediterranean *Cerastoderma glaucum* shells.

Two double-holed greenstone pendants (or buttons) were also found at NEG II. Both are oval-shaped and have a small perforation close to each narrow end. These beads are rare in Natufian sites and have been proposed as a chronological marker of the Late Natufian and early PPNA [50]. Similar greenstone pendants were found at Final Natufian Eynan (e.g. [49, 51]), Late Natufian Gilgal II [52] and the Harifian site of Ramat Harif (GVIII) [53]. Several lines of evidence suggest that these unique ornaments were imported at NEG II: (1) greenstone was not locally available; (2) no production evidence such as waste or preforms were recovered; and (3) only two specimens were found. Thus, unlike the Cardium disc beads which were locally produced these green-stone ornaments were probably traded between Late Natufian groups.

**Fig 15:**
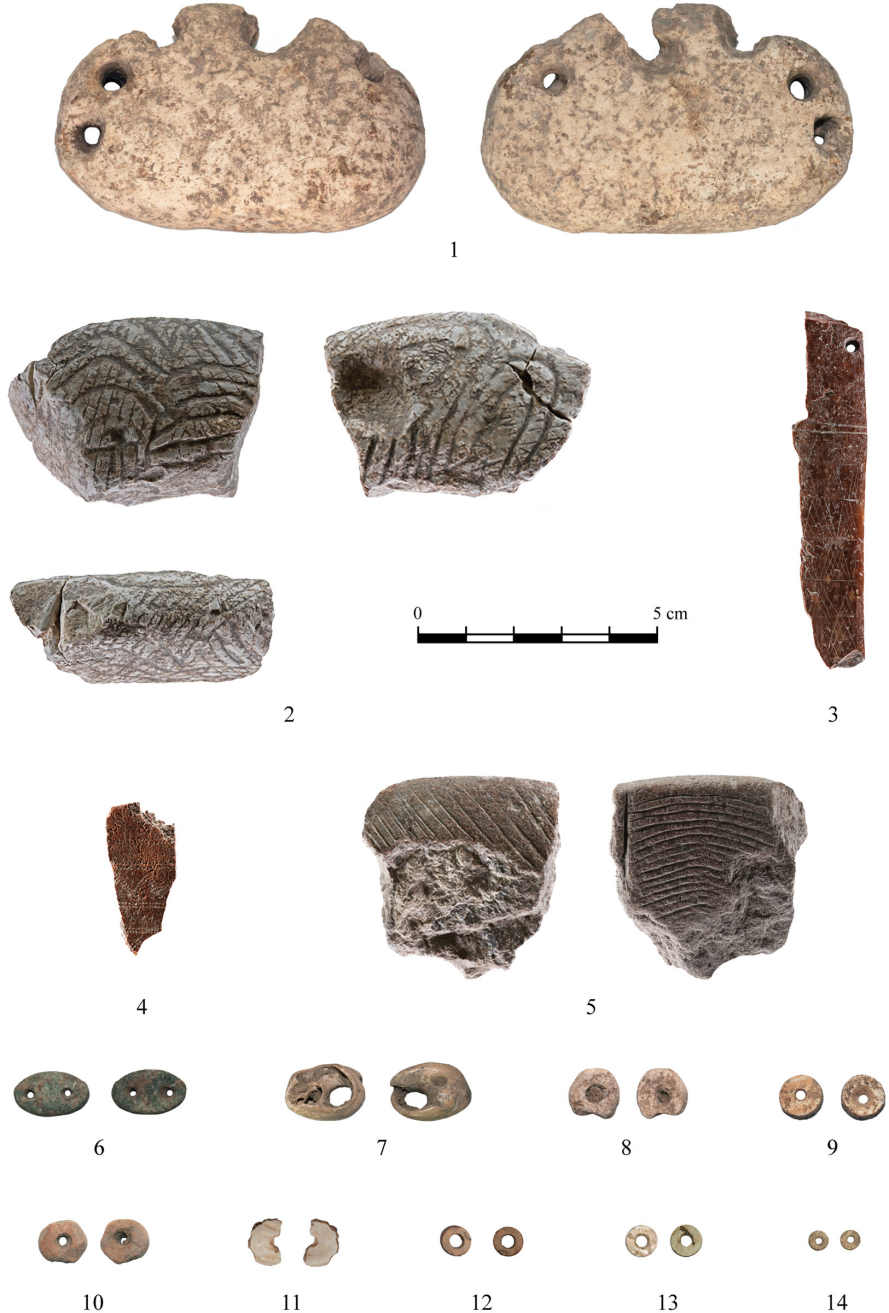
Special Items: 1: Perforated Piece; 2–5: Decorated objects; 6: Green Stone Spacers; 7: Shell Bead; 8–10: Disc Beads; 10, 12–14: Disc Beads Pre-forms. (2–5 photographed by Gabi Laron; 1, 6–14: photographed by Ella Klein).

In addition to the personal ornaments, 20 complete and fragmented art objects were recovered during the 2010–2013 excavations. They include two bone objects (Fig 15) incised with a very delicate and formal repeated geometric pattern of closed triangles bordered by registers. These motifs have previously been observed at Natufian sites but occur much more frequently in the northern Levant PPNA art. The Early Natufian artistic tradition includes antecedents of the NEG II pattern in both, composition—a running pattern of geometric shapes enclosed in registers (e.g., decorated stone bowls from Early Natufian at Eynan; [51]); and motif elements—repeating triangles defined under a line evident in the *festones* pattern on decorated bone objects from Early Natufian Hayonim Cave [3, 54]. Nevertheless, the appearance of the Early Natufian *festones* pattern is restricted by large framed shapes and its basic elements are horizontal lines and repeated notches (triangular notches). In north Levantine PPNA sites similar motifs were applied to various media, including an incised stone plaque (Tell 'Abr 3, [55]) and architectural decorations on communal buildings (e.g. Jerf el-Ahmar, [56]; Tell 'Abr 3, [55]).

Two unique incised stone objects are worth mentioning. The first (Fig 15:2) is a fragment of a limestone object decorated with a linear pattern on all three of its preserved faces. The decorative lines cover the object’s entire surface. The linear patterns are slightly different on each face, but all include deeply incised curved lines overlapped by a second layer of more delicate straight lines. The depth and density of the lines suggest a three-dimensionally carved object, although the decorative approach is two-dimensional. On one face, a wave-pattern was created by a parallel series of horizontally arced lines. On the opposite face a set of curved lines arranged in a vertical-oblique direction transforms the stone surface into a wavy textured relief. The direction of the peak of the arc is altered every few lines. On the third, narrow face, the delicate lines are better preserved than the deep parallel incisions with an oblique orientation.

No exact parallels of this object are currently known from the Late Natufian or PPNA, although similar artistic elements occur in the Early Natufian. For example, there are a few Early Natufian objects which surface are entirely covered with incisions, for example an incised pebble from Hayonim Cave; [3]), although the lines are more delicate and the surface is not prepared (e.g. polished). An Early Natufian limestone fragment from Wadi Hammeh 27 [57] has similar deep incisions, but their coverage is more limited and the composition is less complex and formal. The closest PPNA parallels are decorated stone vessels from the northern Levant (e.g. from Körtik-Tepe; [58]). Like the NEG II examples, the entire surface of these vessels is covered by geometric-linear decoration that transforms the texture of the original surface to one of decorative low relief. Nevertheless, these vessels are distinguished from the NEG II object by other traits such as the limitation of the decoration to the exterior of the vessel and more complex decorative patterns (larger variety of forms and more varied line rhythms). The NEG II object thus falls somewhere between the Early Natufian and northern PPNA examples in terms of the complexity and formality of the decorative linear patterns. It is also distinct in two of its style qualities: (1) the use of two superimposed layers of linear incisions to provide a more elaborate composition and (2) its specific line-form, i.e. low curves.

The second incised stone object is carefully shaped by polish (Fig 15:5). Linear patterns are engraved on both faces—on one there is a series of diagonal lines and on the other a dense group of curved lines that emanates from a slightly deeper straight line. Although the object is broken on three sides, the shape of the preserved edge and the incised patterns suggest that it was an engraved stone plaque, similar to other Natufian/PPNA examples such as an engraved pebble with a chevron pattern from Jeftelik, a north Levantine Early Natufian site ([59]: Fig 7). This pebble was described as having similar dimensions and motifs, but differs in its bi-lateral composition, the use of a double rather than a single line along the axis of symmetry and more complex zigzags composed of lines oriented in several directions. Other comparable examples have simpler compositions, in which straight lines form borders rather than lines of symmetry (PPNA WF16, [60]; and Zahrat al-Dhra’ 2, [57]). Repeating incised lines on a stone from Eynan (Final Natufian level Ib, [49]) reflect a similar style and pattern but differ in the wavy shape of the lines. The low arc pattern of the incised lines from the NEG II object is similar to those on other incised objects from NEG II (e.g. Fig 15:2,3) but rare at other sites. This may reflect a local style variant.

To conclude, art objects from NEG II present a local style with site-specific aspects. They also share inter-site commonalities reflecting intra-cultural connections, including exchange of finished products as well as shared conventions of art creation. In addition, comparative analysis suggests a number of 'style sequences' from Early Natufian precursors through the art of NEG II towards the PPNA art; yet the nature of these trends is varied.

## Discussion

The excavation at NEG II enables us to closely examine the cultural crossroads at the end of the Paleolithic and the beginning of the Neolithic way of life. The absolute dates verify the chronological assignment of NEG II to the Late Natufian. Furthermore, the lithic analysis indicates a Late Natufian tool kit that differed significantly from the succeeding PPNA assemblages, with a complete absence of the latter’s lithic attributes. In contrast, the artistic style from NEG II seems more closely related to the early PPNA world than to the Late Natufian, although it reveals deep roots in Early Natufian tradition. Comparative analysis indicates a number of stylistic trajectories from the Early Natufian to the NEG II Late Natufian and ultimately, the PPNA. For example, the decorative approach becomes more formalized while the media used to depict specific motifs diversifies over time (e.g., from incised stones and bones to architectural elements).

Several other cultural markers from NEG II including burial customs and gazelle hunting strategies show considerable continuity in the Natufian *weltanschauung*. However, a number of socio-cultural changes in intra-site organization, household size and the emergence of distinct public areas are more reminiscent of the early Neolithic cultures to come. Accordingly, the excavation at NEG II reveals that steps toward a new way of life have been taken, but within clearly Natufian life-ways.

### A local Natufian Phenomena

The comparative analysis of the art objects recovered from NEG II reveals a local 'fingerprint' in the manufacture of Cardium disc beads and the particularity of the decoration on two incised stone objects. The Cardium disc beads suggest a standard *chaîne opératoire* for the manufacture of pierced beads indicating more specialized production. This tradition is shared by groups in the Jordan Valley, although beads were produced locally at each site. Local themes also emerge from the NEG II lithic assemblage which is most similar to assemblages from Gilgal II and Huruq Musa, also located in the Lower Jordan Valley (Table 5; [24, 48], Fig 1). Similarities are expressed in the high ratio of perforators, the typology of the sickle blades and the relatively small number of microliths. These aspects of the assemblage are also most similar to those of succeeding PPNA assemblages in the Jordan Valley. Huruq Musa and Gilgal II are located on the western side of the Jordan valley, thus it is unlikely that NEG II represents an eastern Natufian variant, as suggested previously [11]).

**Table 5:** A general breakdown of tool categories of lithic assemblages from Late/Final Natufian sites in the Jordan Valley. Sources: Salibiya, Eynan and Fazael [61], Huruq Musa [48], Gilgal II [24].

	Nahal Ein Gev II	Salibiya I	Mallaha (Eynan) Ic	Mallaha (Eynan) Ib	Fazael IV	Huzuk Musa	Gilgal II
	N = 909	N = 1391	N = 1219	N = 604	N = 1289	N = 92	N = 168
Endscrapers	3.1	4.1	1.5	1.7	2	0	6.5
Burins	4.5	4.7	7.9	3.1	0.5	0	1.8
Perforators	34.1	3.7	2.2	5.3	5.2	38	23.8
Backed pieces	9.4	5.9	3.2	5	10.5	3.3	20.2
Truncations	3.5	2.7	4	4.1	4.2	1.1	2.4
Notches and Denticulates	3.9	4.1	16.1	16.4	5.9	1.1	10.7
Retouched Items	13.9	14.7	7.5	5	12.3	32.6	13.7
Composites	0.6	0	0.7	0.5	0.1	0	3.6
Varia	5.2	2.9	9	17.9	2.8	0	1.2
Non geometric microliths	17.1	42.9	41.6	32.1	39.6	17.4	4.8
Geometric microliths	4.8	14.1	5.9	8.9	16.9	1.1	5.4

Evidence from our excavations suggest that NEG II, Huruq Musa and Gilgal II share elements of both the Late Natufian and the PPNA cultures of the Jordan Valley [24, 62, 63], indicating a local variant of the Late Natufian in this region. Nevertheless, further investigations are needed to fully describe the cultural-chronological identity of NEG II.

### Networks

Connections with groups further afield are suggested by the double-holed green stone pendants that were likely traded across large geographical areas (*i*.*e*. from the southern Negev to the Hula Valley in the north) at the end of the Epipaleolithic period. No greenstone sources have been located close to NEG II [49]. These particular personal ornaments are effective 'chronological markers' of the Late Natufian and PPNA and have no antecedents or exact parallels after the PPNA. The lack of evidence for local manufacture in the Jordan Valley suggests long distance exchange of finished products. Indeed, personal ornaments made from greenstone diversify in form and become one of the key markers of the Neolithic cultures of the Near East [50].

### Was Nahal Ein Gev II a Sedentary Community?

The excavation at NEG II has exposed a complex site plan whose limits still need to be established. To date, the excavated area and the test pits reveal an extensive habitation area covering *ca*. 1200 m^2^ with deep cultural deposits (2.5 to 3 m deep). Although the materials used to build the walls and roofs were not preserved, the remains of several large structures with stone foundations reflect significant investment in permanent architecture. The buildings share several common features including semi-subterranean construction, of 5 m in diameter, basalt and limestone construction materials, with flat stone surfaces facing the interior of the structure, etc. Despite their commonalities, each structure also exhibits unique characteristics such as the building with the bench and the burial in the foundation of the wall of Building 3. The long row of seating provided by the bench may even suggest that the structure served a communal function as a gathering place, heralding a similar structure at PPNA Jerf-el-Ahmar [64]. The buildings may represent several occupation units, whose chronological subdivision will be resolved with further excavation. However, thus far at least four occupational stages are represented at NEG II.

Was this extensive and complex occupation a sedentary village? Sedentism generally refers to a permanent (year round) habitation. Unfortunately, the lack of effective seasonal markers such as house mice or a sufficiently large gazelle tooth sample for mortality profile analysis make it difficult to comment on the seasonality of site occupation at this point (e.g., [65]). Nevertheless, architectural planning, construction methods and various aspects of the faunal assemblage provide good indications for site permanence. The size of the site, the thick archaeological deposits and invested architecture suggest a large, sedentary community at least on the scale of the Early Natufian camps in the Mediterranean zone. Likewise, the uniformity of the flint artifacts (manufacturing and tool types) indicates that at least 2 m of anthropogenic sediments represent intensive occupation of the site by the same cultural entity.

The relative taxonomic abundance of the fauna supports other archaeological data. The ratio of high to low-ranked small game provides an effective measure of site occupation intensity. As humans live longer at a site their impact on local resources increases—particularly on small game taxa which have small home ranges [36]. Humans are expected to preferentially hunt high-ranked tortoises and only turn to lower-ranked prey when tortoises are unable to meet demand. The relative abundance of birds, hares and fish is about equal to that of tortoises. This more closely resembles an Early Natufian than a Late Natufian hunting strategy in the Mediterranean Hills. Fast small game types such as hares and birds are less common at NEG II than in Early Natufian sites in the Mediterranean zone, but significantly more common than in other Late Natufian sites [36, 39]. These data suggest more intensive site occupation at NEG II than at Late Natufian sites in the Mediterranean zone [36, 39]. All in all, the evidence points to NEG II as a permanent site akin to Early Natufian sites in the Mediterranean Hills.

The permanence of settlement is also reflected in the social structure and integrative social mechanisms of the community [6]. Residents of larger villages form more complex social units that extend beyond close kin [66]. From the Early Natufian to the PPNA social construction involved the establishment of ritually charged spaces such as cemeteries [67, 68]. In some cases this ritual intensification took place within individual households rather than in newly constituted public spaces. At the Natufian village of NEG II the burial of the dead was integrated into individual households but there was at least one designated burial area (Area A) that will be further exposed during future excavation.

### Population Mobility and the Younger Dryas

NEG II was occupied in the midst of the cold and dry global climatic event known as the Younger Dryas (YD, 12,900–11,600 cal BP). Despite solid evidence for the YD in diverse climatic datasets from northern latitudes, the scale of its impact in the Levant has been debated. A recent review of multiple datasets reveals that the YD in the Jordan Valley was not as arid as thought ([69, 70] and references within). In fact, annual rainfall was likely similar to that of present day. Likewise, recent data from gazelle inhabiting the Mediterranean zone of the Galilee region in the YD suggest that rainfall remained steady across the YD, although temperatures cooled [71]. Thus it seems that with the exception of cooling in the Mediterranean zone, the impact of the YD in the Natufian ‘core area’ especially the Jordan Valley, was not as extreme as originally believed [72]. The archaeological sequence in the ‘core area’ suggests an earlier gradual transition to the Late Natufian.

The scale of impact of the YD is important, because archaeological evidence suggests that the local inhabitants who were nearly sedentary in the Early Natufian in the Mediterranean zone [6], reverted to a more nomadic way of life in the Late Natufian, and that most Late Natufian sites, such as Eynan, represented short-term occupations of small hunter-gatherer bands [7, 12, 39]. This shift in settlement pattern may have been a response to changes in biomass productivity brought on by cooling induced by the YD in the Mediterranean zone [12, 71]. Lower densities of key resources, namely cereal grains would reduce the length of time that hunter-gatherers could stay in a particular location without exhausting local resources.

Although small-scale, short-term climatic oscillations must have occurred, [73], the scale of the archaeological and architectural evidence from NEG II correlates with the new climatic data for the YD in the Jordan Valley. Unlike the increasingly mobile Late Natufian residents of the Mediterranean zone where conditions appear to have been cooler, the occupants at NEG II inhabited a more or less permanent village even during the YD. This contrast may be due to greater climatic stability in the Jordan Valley, that allowed larger-scale, permanent settlements to survive in this area.

In the past it was suggested [3, 7, 74, 75] that the change in site function and increased population mobility in the Mediterranean Hills during the Late Natufian also reflects the failure of cultural mechanisms to maintain large communities on a permanent basis. Nevertheless, recent evidence suggests that Late Natufian bands preserved ancestral cultural traditions using ritual practice as a powerful device for social cohesion [74]. It has been proposed that to promote group unity and eradicate budding social stratification and cultural differentiation between kin-groups, the Late Natufians sought to treat all community members as equals (e.g., [66]regarding mortuary practices). Thus although previous arguments conclude that the YD climatic event interrupted the cultural dynamics that promoted the shift to agriculture, archaeological evidence from NEG II show that the trend toward the establishment of farming communities maintained its course throughout the Late Natufian phase. Likewise, in the Jordan Valley, the Late Natufian culture portrays a complex cultural tradition typical of large sedentary communities.

In summary, the site of NEG II does not conform to current perceptions of the Late Natufians as mobile populations coping with reduced resource productivity and cultural continuity caused by the YD. It seems that the Natufians, who started out as sedentary in the Early Natufian, did not revert, at least in the Jordan Valley, to a nomadic way of life in the Late Natufian. The sub-phases and the dense occupation at NEG II suggest a large, intensive village occupation that endured for more than a few centuries. The area dedicated to human burial portrays a lasting connection between the living and the dead of the community.

The evidence from NEG II reveals a shift in settlement pattern in the Late Natufian. While Mediterranean groups became increasingly mobile and potentially smaller in size, those in the Jordan Valley became even more sedentary and potentially larger in size. This may be related to greater climatic stability, higher cereal biomass productivity and better conditions for small-scale cultivation that provided the ingredients necessary to take the final steps toward agriculture in the southern Levant. It is not surprising that at the very end of the Natufian culture, it is at a suite of sites in the Jordan Valley that we find a cultural entity that bridges between Late Paleolithic foragers and Neolithic farmers.
